# Promoter activity of Merkel cell Polyomavirus variants in human dermal fibroblasts and a Merkel cell carcinoma cell line

**DOI:** 10.1186/s12985-020-01317-x

**Published:** 2020-04-19

**Authors:** Ibrahim Abdulsalam, Kashif Rasheed, Baldur Sveinbjørnsson, Bernhard Ehlers, Ugo Moens

**Affiliations:** 1 Molecular Inflammation Research Group, Department of Medical Biology, Faculty of Health Sciences, University of Tromsø, The Arctic University of Norway, Tromsø, Norway; 2 Present address: Tumor Biology Research Group, Department of Medical Biology, Faculty of Health Sciences, University of Tromsø, The Arctic University of Norway, Tromsø, Norway; 3 Division 12 Measles, Mumps, Rubella and Viruses Affecting Immunocompromised Patients, Robert Koch Institute, Berlin, Germany

**Keywords:** Non-coding control region, Large T-antigen, Luciferase assay, MCC13 cells, MCPyV, Mutations

## Abstract

**Background:**

Merkel cell polyomavirus (MCPyV) is a human polyomavirus that establishes a life-long harmless infection in most individuals, with dermal fibroblasts believed to be the natural host cell. However, this virus is the major cause of Merkel cell carcinoma (MCC), an aggressive skin cancer. Several MCPyV variants with polymorphism in their promoter region have been isolated, but it is not known whether these differences affect the biological properties of the virus.

**Methods:**

Using transient transfection studies in human dermal fibroblasts and the MCC cell line MCC13, we compared the transcription activity of the early and late promoters of the most commonly described non-coding control region MCPyV variant and six other isolates containing specific mutation patterns.

**Results:**

Both the early and late promoters were significantly stronger in human dermal fibroblasts compared with MCC13 cells, and a different promoter strength between the MCPyV variants was observed. The expression of full-length large T-antigen, a viral protein that regulates early and late promoter activity, inhibited early and late promoter activities in both cell lines. Nonetheless, a truncated large T-antigen, which is expressed in virus-positive MCCs, stimulated the activity of its cognate promoter.

**Conclusion:**

The promoter activities of all MCPyV variants tested was stronger in human dermal fibroblasts, a cell line that supports viral replication, than in MCC13 cells, which are not permissive for MCPyV. Truncated large T-antigen, but not full-length large T-antigen stimulated viral promoter activity. Whether, the difference in promoter strength and regulation by large T-antigen may affect the replication and tumorigenic properties of the virus remains to be determined.

## Background

In 2008, a new human polyomavirus was isolated, which rekindled the field of polyomavirus research [1]. This virus was isolated from Merkel cell carcinoma (MCC), a rare but aggressive skin cancer. Accordingly, this virus was named Merkel cell polyomavirus (MCPyV). The original study showed that 8 out of the 10 examined MCC samples contained MCPyV DNA [1]. Numerous studies by different groups worldwide have confirmed that approximately 80% of MCCs are positive for this virus [2–5]. Because cell culture and transgenic mice studies have shown that MCPyV has an oncogenic potential that can be attributed to its viral proteins large T-antigen (LT) and small t-antigen (sT) ([6–9]), and the association of the virus with MCC, MCPyV is considered an etiological factor in MCC and is classified as probably carcinogenic to humans [10]. Two hallmarks of MCPyV-positive MCCs are the integration of the viral genome in the host chromosome and expression of a truncated version of LT [5, 11]. Integration disrupts the late region so that no infectious particles are generated in MCCs, while the truncation of LT results in a non-DNA binding variant that retains the ability to bind the tumor suppressor retinoblastoma protein, but not p53 [12].

Serological studies demonstrated that seroprevalence against MCPyV increases with age, and reaches up to ~ 80% in healthy individuals [13–18]. Little is known about the route of infection, transmission and the cell tropism of MCPyV. Dermal fibroblasts are a genuine host cell for MCPyV [19], and the virus seems to persist in the skin [20–22]. However, PCR-based analyses detected MCPyV DNA in other sites in the body, both in healthy individuals and patients (Supplementary Table S1), as well as in sewage water and environmental surfaces (Supplementary Table S2). The implication of MCPyV in cancers other than MCC remains unknown, although viral DNA, RNA and proteins can be detected in some cases of other malignancies [23]. Sequence analysis of the MCPyV LT, sT and VP1 genes of different virus isolates revealed genetic variability, but the biological implications in the viral life cycle and the development of MCC have not been studied.

Mutations in the non-coding control region (NCCR) of human polyomaviruses like BKPyV, JCPyV, KIPyV, HPyV7, HPyV9 and HPyV12 have an impact on the transcriptional activity of the promoter, and may affect the virulence of the virus [24–33]. Whether changes in the NCCR of MCPyV have an effect on the promoter activity, and have pathogenic consequences, has not been investigated. Here, we compare the transcriptional activity of NCCR of different MCPyV variants isolated from virus-positive MCC and non-MCC samples in a MCC cell line, and in human dermal fibroblasts.

## Methods

### Cells

The MCPyV-negative MCC13 cell line was kindly provided by Dr. Baki Akgül (University of Cologne, Germany) and was grown in RPMI-1640 (Sigma Life Science, St. Louis, MO. USA; cat. no. R8758) with 10% fetal bovine serum (Gibco, Life Technologies Limited, Pailey, UK) in the presence of 100 µg/ml streptomycin and 100 units/ml penicillin. Immortalized human dermal fibroblasts fHDF/TERT166 were purchased from Evercyte (Vienna, Austria) and kept in DMEM/Ham’s F12 (1:1) (Biochrom, Berlin, Germany; cat. no. F4815), 10% fetal bovine serum, 2 mM GlutaMaxTM-I (Giboco; cat. no. 35050–038) and 100 µg/ml G418 (Santa Cruz Biotechnology, Dallas, TX, USA; cat. no. sc-29,065). Cells were kept in a humidified CO_2_ incubator at 37 °C.

### Plasmids

The luciferase reporter plasmids with the consensus NCCR MCPyV in early (pGL3-cons-E) or late (pGL3-cons-L) orientation have been previously described [24]. The luciferase reporter plasmids containing the NCCR of the variants 10b, 15a, 16b, HUN, MKL-1, MS-1 were generated by GenScript (Piscataway, NJ, USA). Each NCCR was cloned in both early (NCCR-E) and late (NCCR-L) orientation, respectively. The luciferase plasmids with the NCCR containing the 25 bp duplication described by Hasida et al. [34] was generated by site-directed mutagenesis using the plasmid pGL3-cons-E (pGL3-cons-L, respectively) containing the consensus NCCR and the complementary primers 5′- GGCCGGAGGCTTTTTTTTCTCTTACAAAGGGAGGAGGACATTTCTCTTACAAAGGG-3′ and 5′-CCCTTTGTAAGAGAAATGTCCTCCTCCCTTTGTAAGAGAAAAAAAAGCCTCCGGCC-3′. The empty expression vector pcDNA3.1(+) was purchased from Invitrogen (ThermoFisher Scientific, Oslo, Norway). The MCPyV expression vectors for full-length and truncated LT have been previously described [35], and all plasmids were verified by sequencing. Expression of full-length and truncated LT was confirmed by western blotting using antibody CM2B4 from Santa Cruz Biotechnology (Dallas, TX, USA; cat. no. sc-136,172; results not shown).

### Transfection and luciferase assay

Cells were seeded out in 12-well culture plates. At the time of transfection, the cells were approximately 70% confluent, with a total of 1 µg luciferase reporter plasmid DNA used per well and polyethylenimine (PEI linear MW25000; transfection grade, cat. no. 23966–1, Polysciences, Warrington, PA, USA). DNA was mixed with 150 mM NaCl, and a mixture of PEI:150 mM NaCl was then added to the DNA. The ratio DNA:PEI used was 1:2. This mixture was incubated for 15 min at room temperature, and then carefully added to the cells. The medium containing the transfection mixture was replaced 4 h later. Cells were harvested 24 h after transfection in a 100 µl Tropix lysis buffer per well with 0.5 mM DTT freshly added. Cells were centrifuged for 3 min at 12,000 g, and the supernatant was then transferred to a fresh tube. As previously described, 20 µl of supernatant was used in the luciferase assay [35]. Each experiment was repeated at least 3 times, with three independent parallels for each experiment. Luciferase values for each sample were corrected for total protein concentration as determined with the MN protein quantification assay described by the producer (Macherey-Nagel GmbH, Düren, Germany). We corrected luciferase values by measuring the protein concentration in the corresponding sample rather than co-transfection with a Renilla reporter plasmid to avoid promoter interference between the MCPyV NCCR directing expression of the firefly luciferase gene and a promoter controlling expression of the Renilla luciferase gene. In addition, many of our transfection studies include co-transfection with LT expression plasmids, containing the strong competing CMV promoter. Moreover, LT of polyomaviruses have shown activate many promoters, including the SV40 promoter or the herpes simplex virus thymidine kinase promoter [36], which are commonly used in Renilla reporter plasmids.

### Statistics

A two-tailed Student’s *t*-test was used to determine statistical differences between the MCPyV promoter variants.

## Results

### The known MCPyV NCCRs can be classified in six different groups

**Fig. 1:**
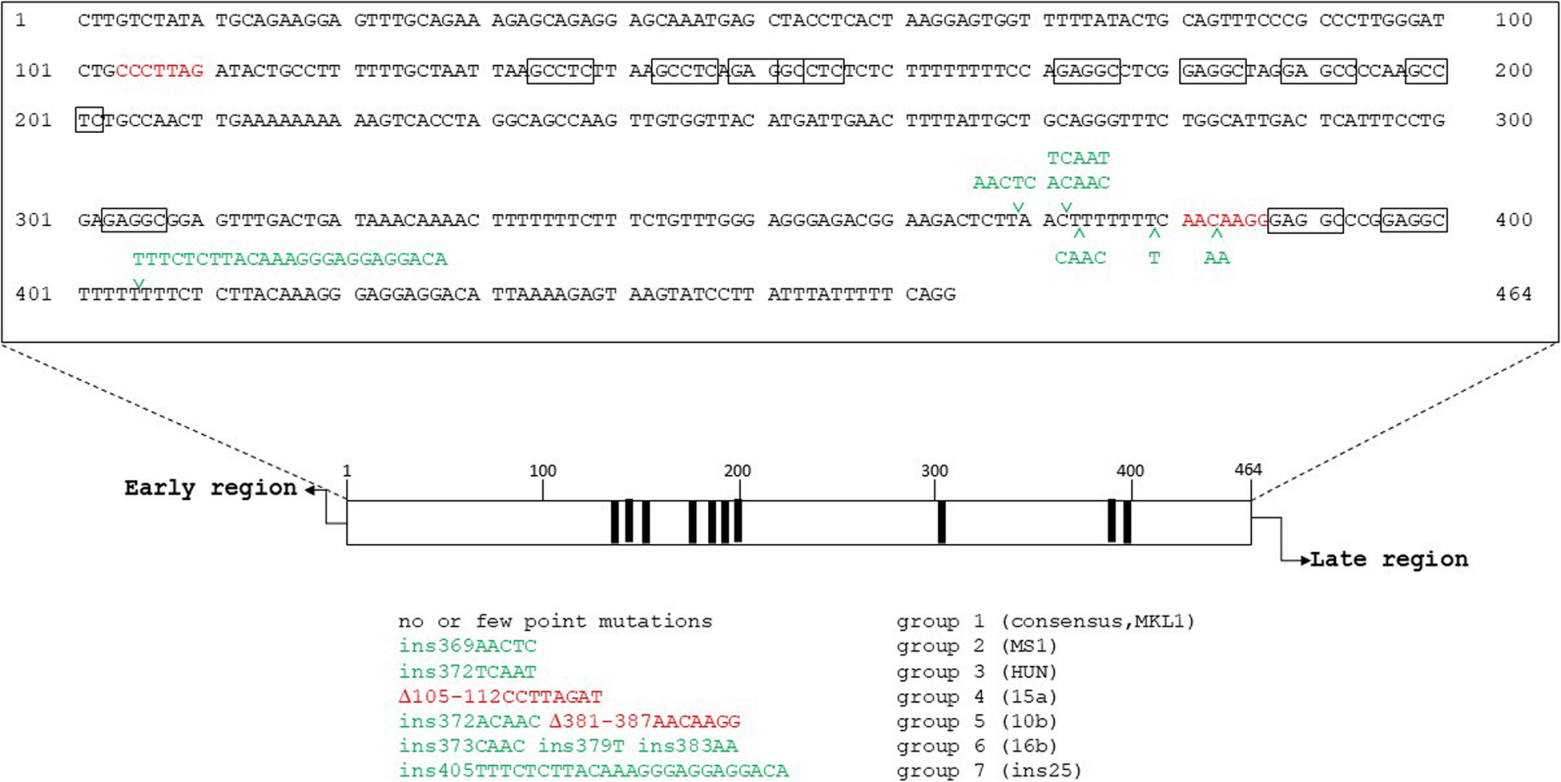
The MCPyV NCCR region and the different variants. The top panel of the figure shows the consensus nucleotide sequence based on variant R17b (GenBank accession number NC_010277), with the first nucleotide in the NCCR numbered 1 and the last numbered 464. The putative LT binding sequences (GRGGC) are shown in boxes. The bottom part shows a schematic presentation of the NCCR, with the thick vertical lines representing putative LT binding motifs [37]. The early region is indicated on the left and the late region on the right. The different groups of NCCR variants and their major mutations are indicated. The larger insertions (ins) and deletions (Δ) are given, whereas point mutations are not shown

A comparison of all available complete NCCR sequences of MCPyV variants revealed a predominant sequence, which is hereafter referred to as the consensus sequence shown in Fig. 1. Based on this consensus sequence, we classified the different NCCR variants in seven groups (Fig. 1 and Table 1). Group 1 contains the MCPyV strains with a consensus or quasi consensus (i.e. one or few point mutations). Group 2 contains NCCR variants with an insertion of the AAC or AACTC sequence at nucleotide 369 (numbering according to the consensus sequence). Group 3 NCCR has an insertion of the TCAAT sequence at nucleotide 372, while group 4 has deletion of the CCTTAGAT sequence (nucleotides 105–112). Group 5 has both an insertion (ACAA or ACAAC at nucleotide 372) and a deletion of nucleotides 381–387 (AACAAGG). The NCCR in group 6 has three insertions: CAAC after nucleotide 373, T after nucleotide 379 and AA after nucleotide 383. Lastly, group 7 variants have a 25 bp duplication.

**Table 1:** MCPyV NCCR variants examined in this study

Group	NCCR variant	Referred to in this paper	Source	Reference
1	R17b	cons-E and cons-L	healthy skin	[20]
	MKL-1	MKL1-E and MKL1-L	MCC	[38]
2	MS-1	MS1-E and MS1-L	MCC	[1]
3	7673/2011/HUN	HUN-E and HUN-L	metastatic cervical lymph node	[39]
4	R15a	15a-E and 15a-L	healthy skin	[20]
5	R10b	10b-E and 10b-L	healthy skin	[20]
6	R16b	16b-E and 16b-L	healthy skin	[20]
7	Subtype I	Ins25-E and ins25-L	healthy skin	[34]

The biological source of each NCCR variant is given in Table 1 and Supplementary Table S1. Consensus NCCRs are found in strains present in non-diseased and diseases tissue/individuals. Likewise, NCCR variants circulate in healthy individuals and patients. MCPyV variants with consensus and mutated NCCRs have been isolated from sewage water (see Table 1 for references). The mutations for each NCCR variant are presented in Supplementary Table S3.

### Basal early and late promoter activities in MCC13 and human dermal fibroblasts cells

Because MCPyV-positive Merkel cell carcinoma derives from virus-transformed Merkel cells [1], and human dermal fibroblasts (HDF) have been shown to be permissive for this virus and considered as genuine host cells for the virus [19], we examined the basal early and late promoter activity of MCPyV variants in these two cell lines. To study the effect of mutations in the NCCR on basal early and late promoter activity, one NCCR variant was selected from each group, with the exception of group 1, in which both the consensus sequence and a variant with few point mutations were tested (Table 1). The NCCRs were cloned in both orientations, and the basal early and late promoter activities were monitored in the MCPyV-negative MCC cell line MCC13, in addition to primary dermal fibroblasts.

Comparing the relative basal early and late promoter activities showed that both promoters were stronger in HDF cells compared to MCC13 cells (Supplementary Fig. S1). The cons-E promoter was ~ 4-fold stronger, while the cons-L was ~3x stronger. The difference is probably even more because the transfection efficiency in MCC13 cells was approximately 60–70%, whereas in HDF the transfection efficiency was estimated to be ~ 30% (results not shown). Comparing the cons-E and cons-L in MCC13 revealed that the late promoter was approximately 8x stronger than the early promoter. The cons-L was approximately 6x stronger in HDF than in MCC13 cells (Supplementary Fig. S1).

### Effect of large T-antigen on early and late promoter activities

Large T-antigen of polyomaviruses has been shown to affect the early and late promoter activities. To examine the effect of LT on the MCPyV promoters, we co-transfected MCC13 cells with 1 µg luciferase reporter plasmid with the cons-E promoter, with increasing amounts (0, 100, 200, 400, 500, 800 and 1000 ng) of empty pcDNA3.1 vector or LT expression vector. All concentrations reduced cons-E promoter activity (results not shown), but high concentrations (800 and 1000 ng) of empty vector almost completely inhibited MCPyV promoter activity. Both the empty vector and the LT expression plasmid contain the strong CMV immediate early promoter. We found this promoter to be > 5-fold stronger than the cons-E promoter in MCC13 cells and ~40x stronger in HDF cells (Supplementary Fig. S2). We decided to test the effect of two different concentrations (100 and 500 ng) of LT expression plasmid on all variant NCCRs. Both concentrations of LT expression plasmid (100 ng and 500 ng) significantly reduced the activity of all early promoters in MCC13 cells (Fig. 3a and b). A similar effect was observed for the late promoters, with the exception of the MS1 and HUN promoters, which were induced when cells were co-transfected with 500 ng of LT expression plasmid (Fig. 3c and d).

We also examined the effect of LT on the MCPyV promoters in HDF cells. Both concentrations of LT expression plasmid (100 ng and 500 ng) significantly repressed the early promoter activity (Fig. 4a and b) and the late promoter activity (Fig. 4c and d) of all variant NCCRs tested. However, a somewhat stronger inhibition was observed with the lowest concentration of LT expression plasmid, while at higher concentrations promoter interference becomes more pronounced.

MCPyV-positive MCCs contain integrated viral DNA, and are further characterized by the expression of a C-terminal truncated LT [Feng, 2008]. Thus, we examined the effect of truncated LT on the early and late promoter. Of the seven different strains examined in this work, only MKL-1 and MS-1 have been isolated from MCC [40, 41], whereas the HUN strain was obtained from a metastatic cervical lymph node from a Hungarian patient, though no further information is available [39]. We therefore decided to test the impact of MKL-1 (MS-1, respectively) truncated LT on their cognate promoter. The luciferase reporter plasmids with the early or late promoter of MKL-1 (MS-1, respectively) were co-transfected with an expression plasmid for MKL-1 LT (MS-1 LT, respectively) in MCC13 and in HDF cells, and the promoter activity was monitored. Truncated MKL-1 LT stimulated the MKL-1 early and late promoter activities in both MCC13 and HDF cells (Fig. 5a and b). The only exception was when 500 ng of MKL-1 LT expression plasmid was used, with a significant inhibition of the early promoter observed in HDF cells (Fig. 5b). Truncated MS-1 LT also stimulated the early and late MS-1 promoter in MCC13 cells and the late promoter in HDF cells (Fig. 5a and b), but inhibited the early promoter in HDF cells (Fig. 5b). With 100 ng of truncated MS-1 LT expression plasmid, a reduction in promoter activity of 15–24% compared to the control was observed. However, this decrease was not significant (*p*-values between 0.0553 and 0.0632 in the different experiments).

## Discussion

MCPyV has a seroprevalence of approximately 80% in the healthy, adult population [13–18]. MCPyV is chronically shed from healthy skin [20], but can also cause an aggressive skin cancer known as Merkel cell carcinoma [1]. Several MCPyV variants have been described with mutations in their NCCR (Supplementary Table S3). These variants have been isolated from both healthy tissue and tumors, but so far, no typical strain seems to be associated with MCC (Supplementary Table S3). The mutations described in the known MCPyV variants could be classified in seven groups with group 1 containing the most common NCCR, which was referred to as the consensus sequence in our study and variants with one or a few point mutations. Groups 2–7 contain insertions and/or deletions in their NCCR. Significant differences in basal early and late promoter activities in HDF and MCC13 cells were observed. The basal early-, as well as late promoter activity, of all variants tested was higher in HDF cells than in MCC13 cells despite a lower transfection efficiency (Fig. 6). This may indicate that the MCPyV promoter is more adapted to the former cell type. HDF have been suggested as natural host cells for the virus and are permissive for the virus, while the infection of Merkel cells and the subsequent transformation of these cells could be seen as an accidental and unfortunate event [19].

Co-transfection with a 100 ng of full-length LT expression plasmid resulted in reduced early and late promoter activity of all variants in both MCC13 and HDF cells (Fig. 6). However, 500 ng of LT expression plasmid reduced early and late promoter activities in HDF and early promoter activity in MCC13 cells, but had only a slight or no effect, but significantly stimulated the HUN and MS1 (a 20 and 40% increase, respectively) late promoters. Kwun et al. found that MCPyV LT repressed early and late promoter activity of MCPyV isolate MCC339 in HEK293 cells, but they did not examine the effect of LT in MCC13 or HDF cells [42]. Yet, in another study by Ajuh and co-workers, the authors showed that LT trans-activated the MCPyV R17b (=consensus) early and late promoter in HEK293T cells [29]. The discrepancy between their results and the findings by Kwun et al. and us can be explained by the use of different LT. HEK293T cells express both SV40 LT and sT, but not MCPyV LT [29]. Moreover, the possible contribution of sT in the trans-activation of the early and late MCPyV promoters in these cells was not investigated. Furthermore, Ajuh et al. studied the effect of LT using a bidirectional reporter vector, thereby allowing for the simultaneous monitoring of the early and late promoter activity, whereas both we and Kwun et al. examined early and late promoter independently, which better reflects the situation in infected cells. Indeed, during the polyomavirus life cycle, early and late promoters are activated in a time-dependent fashion. The early promoter is active early during infection, and the expression of LT will result in the autorepression of the early promoter and a switch to activation of the late promoter [43]. Another experimental difference was that we measured promoter activities 24 h after transfection, whereas Ajuh and colleagues determined promoter activities 48 h post transfection. Lastly, we used dose-dependent studies with LT, while Ajuh and co-workers used cells constitutively expressing LT. The authors also examined the effect of MCPyV LT on early and late promoter activity of the consensus MCPyV variant and MCVw156 (consensus with one substitution and one deletion; Supplementary Table S3) in HEK293MCT cells (i.e. HEK293 cells stably expressing MCPyV LT). While early promoters of both MCPyV variants were significantly stimulated by MCPyV LT, no effect was observed on their late promoter. A possible explanation for the different effects of SV40 LT and MCPyV LT on the MCPyV late promoter was not provided by the authors.

Because the viral genome in MCC expresses a truncated LT, we examined the effect of truncated LT on its cognate promoter. Truncated MKL-1 (MS-1, respectively) LT stimulated its cognate early and late promoter in MCC13 cells, whereas stimulation was only observed for the late promoter in HDF cells. In these cells, truncated MKL-1 and MS-1 LT inhibited the corresponding early promoter. The reason for the cell-specific effect of truncated LT on the MCPyV promoter is not known, but MCPyV LT has been shown to interact with several cellular factors [42]. Different interaction partners in distinct cell types may determine the effect of LT on the MCPyV promoter activity. The fact that truncated LT stimulates their cognate early promoter in MCC13 may indicate a positive feedback loop that results in higher expression levels of the early proteins, including the oncoproteins sT and LT. Our preliminary results shows that also truncated MKL-2 LT (which differs from HUN truncated LT by replacement of Ala20 into Ser and Ser263 into Phe, and the lack of the three C-terminal amino acids Ser-Arg-Lys) was able to stimulate the HUN-E promoter approximately 2-fold in MCC13 cells (our unpublished results), suggesting that it may be a common feature of truncated LT to autostimulate its expression. This positive autoregulation of LT and sT could be potentially important for tumorigenesis. Since full-length LT had an inhibitory effect on the promoters, it would be interested to test whether full-length LT reverses the activity of truncated LT. However, to be of biological relevance, both full-length and truncated LT must be co-expressed in MCPyV infected cells. To our best knowledge, only truncated LT is expressed in virus-positive MCCs. We are aware of only two studies were co-expression of full-length and truncated LT was observed. One case of non small cell lung cancer (a squamous cell carcinoma) with both episomal and integrated viral DNA and both full-length and truncated LT protein was described by Hashida and co-workers [44]. In another study, mRNAs for truncated and full- length LT were confirmed by highly sensitive qRT-PCR in two cases of chronic lymphocytic leukemia, but expression of truncated and full-length LT at protein level was not investigated [45]. The genome copy per chronic lymphocytic leukemia cell was 3 to 4 logs lower than MCPyV-positive MCCs, suggesting that very low levels of LT/truncated LT are present in these cells. It remains to be determined whether such low levels have any biological relevance for the viral life cycle or tumorigenesis. Because integration of the MCPyV genome interrupts the late region, no late proteins are expressed and no viral particles are produced. The biological implication of enhanced late promoter activity by truncated LT in MCC remains elusive.

**Fig. 2:**
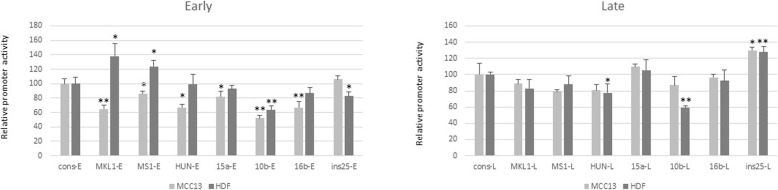
Relative promoter activities of different MCPyV NCCR variants in MCC13 and human dermal fibroblast cells. Cells in 12-well plates were transiently transfected with 1 μg luciferase reporter plasmid containing the early promoter of MCPyV. Luciferase activity was corrected for total protein concentration of the sample, and the corrected value for the early consensus (late consensus, respectively) promoter was arbitrarily set as 100%. Each bar represents the average of three independent parallels + standard deviation. A representative result is shown, and each promoter was tested at least three times in independent experiments with similar results; *p < 0.05, **p < 0.01

Our transient transfection studies showed that the MCPyV NCCR variants possess different promoter activity, and can lead to different expression levels of the viral proteins. This has been confirmed by in situ studies. The CVG-1 and MKL-1 MCC cell lines both contain seven copies of the integrated virus genome per diploid cell [46]. The CVG-1 cell line contains the consensus NCCR sequence, while MKL-1 contains one single point mutation (T52C; Supplementary Table S2). Quantitative real-time PCR demonstrated that the total LT and sT mRNA expression levels were approximately 2.5 times higher in CVG-1 cells compared to MKL-1 cells, thus indicating that the former promoter is 2.5x stronger than the latter. Our transient transfection study in MCC13 cells confirmed that the MKL-1 early promoter was weaker than the consensus early promoter (Fig. 2). The expression levels of early proteins in MCC not only depend on the strength of the early promoter, but the number of integrated viral genomes and the integration site (hetero- versus euchromatin) may also influence the promoter strength. Velásquez et al. determined that MKL-2 and MS-1 MCC cells contain two and four genome copies per diploid cells, respectively [46]. The LT transcript levels were approximately 2-fold higher in MS-1 cells compared to the MKL-2 cells, while the ST mRNA levels were 4x higher in MS-1 cells than in MKL-2 cells. The complete NCCR sequence of the MKL-2 variant has not been determined, but the 240 nucleotides downstream of the LT start codon are identical with the consensus sequence.

**Fig. 3:**
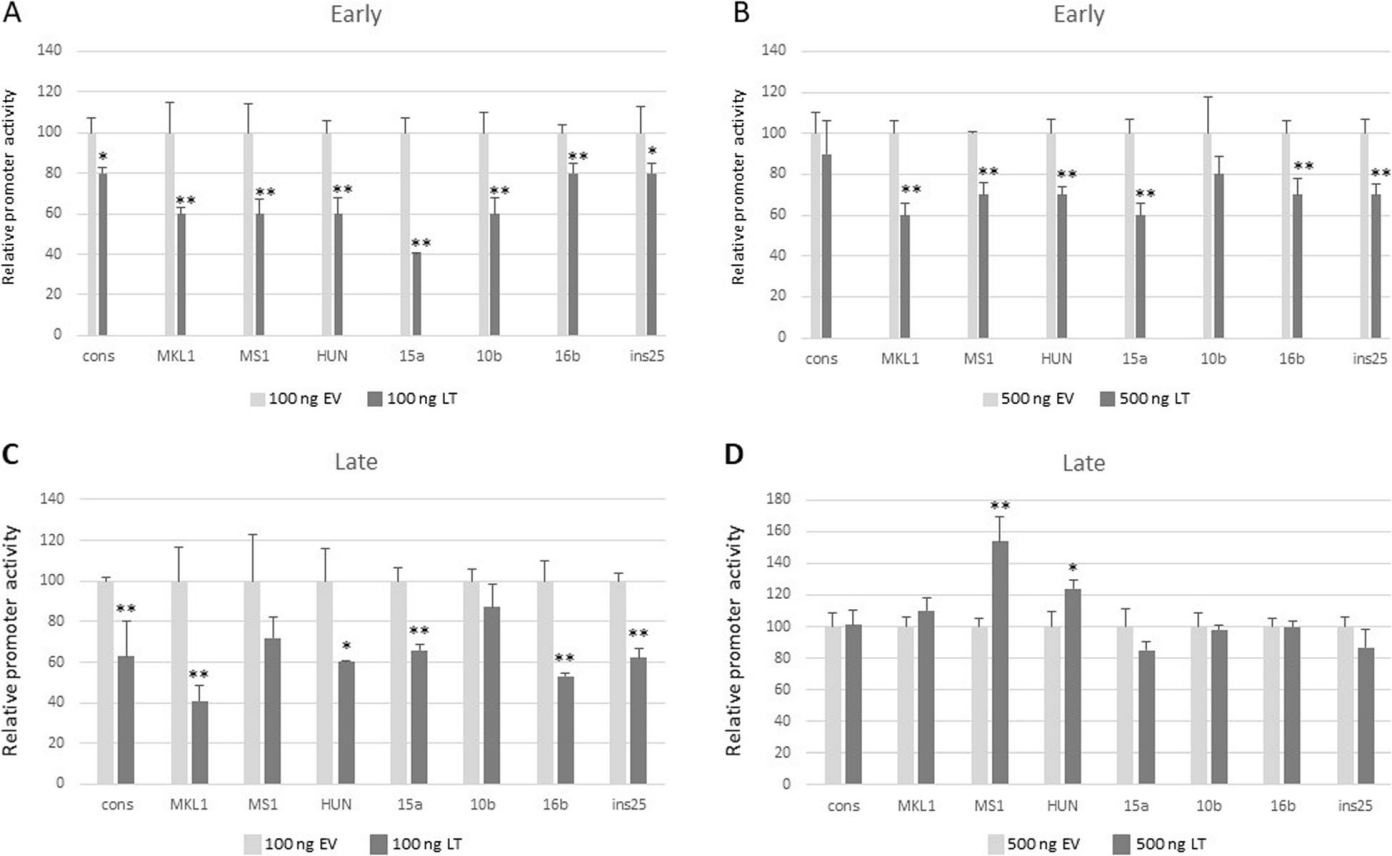
Effect of MCPyV LT on early and late promoter activity in MCC13 cells. Cells were co-transfected with 1 μg luciferase reporter plasmid with the early promoter of the MCPyV variants and 100 ng of empty pcDNA3.1 vector (EV) or LT expression plasmid (**a** and **c**), or with 500 ng of empty vector or LT expression plasmid (**b** and **d**). The effect on the early MCPyV promoters is depicted in A and B, while the effect on the late promoters is shown in C and D. Luciferase activity was corrected for protein concentration in each sample, and the activity in the presence of an empty vector was arbitrary set as 100%. Each bar represents the average of three independent parallels + SD. Similar results were obtained in an independent experiment; *p < 0.05; **p < 0.01

**Fig. 4:**
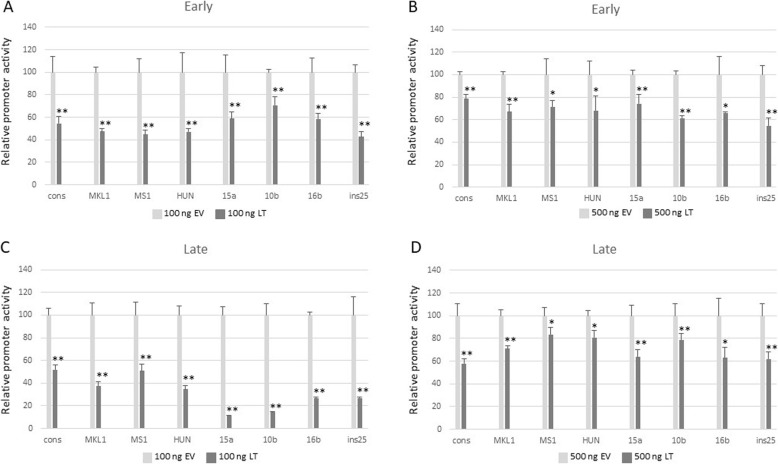
Effect of MCPyV LT on early and late promoter activity in HDF cells. For details, see legend of Fig. 3

**Fig. 5:**
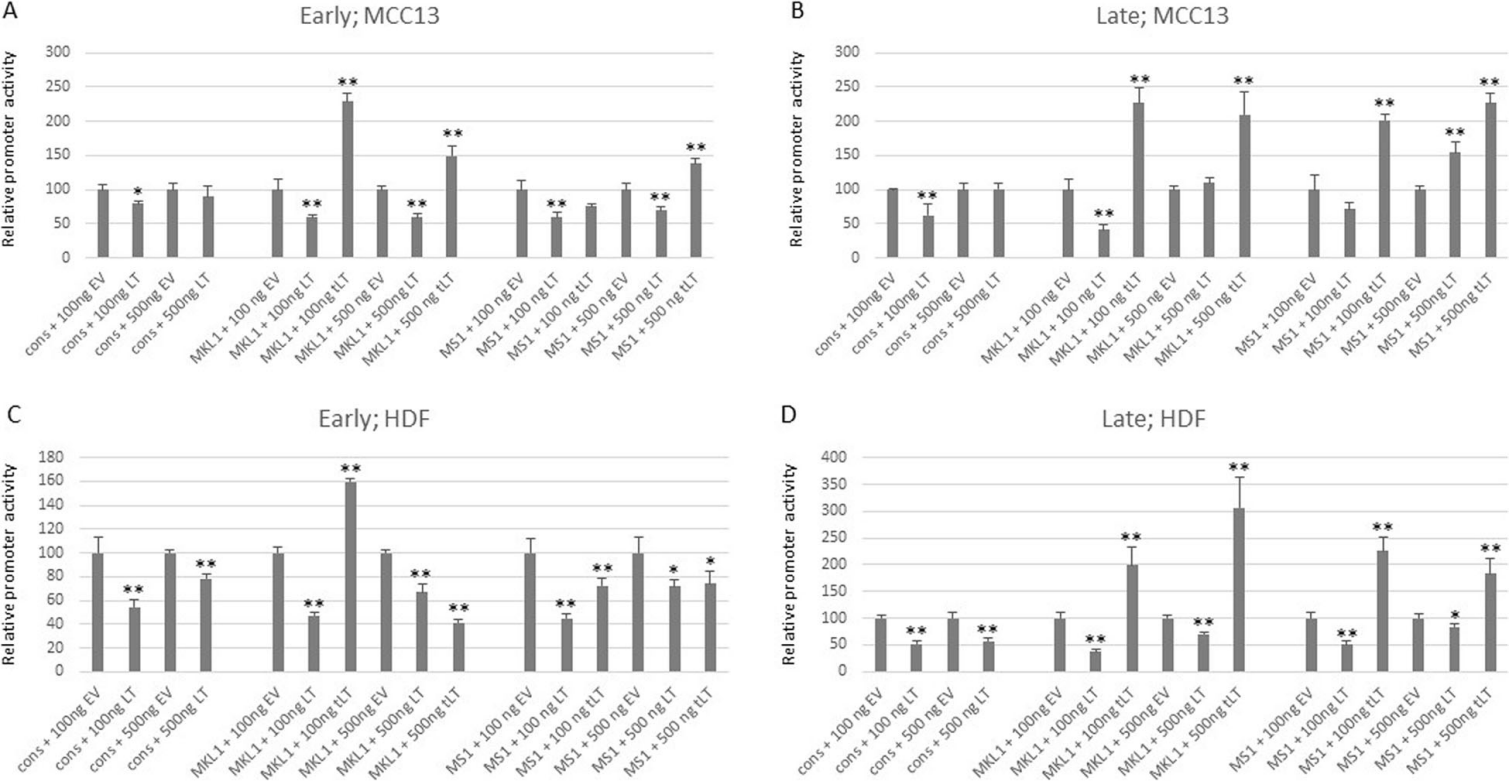
The effect of truncated LT on its cognate promoter in MCC13 and HDF cells. (A) MCC13 cells were co-transfected with the luciferase reporter plasmid containing the early MKL-1 (MS-1, respectively) promoter, and either empty vector (EV), full-length LT, or truncated LT (tLT) encoded by the MKL-1 (MS-1, respectively) MCPyV variant; (**b**) as (**a**,) but luciferase reporter plasmids containing the late promoter of MKL-1 or MS-1 were used. Cells were also co-transfected with the reporter plasmid containing the early or late consensus promoter and empty vector or LT expression plasmid. (**c**) and (**d**) As (**a**) and (**b**), respectively, but transfections were performed in HDF cells. Luciferase activity was corrected for the protein concentration in each sample, with the activity in the presence of the empty vector arbitrarily set as 100%. Each bar represents the average of three independent + SD. Similar results were obtained in two additional experiment; *p < 0.05, **p < 0.01

**Fig. 6:**
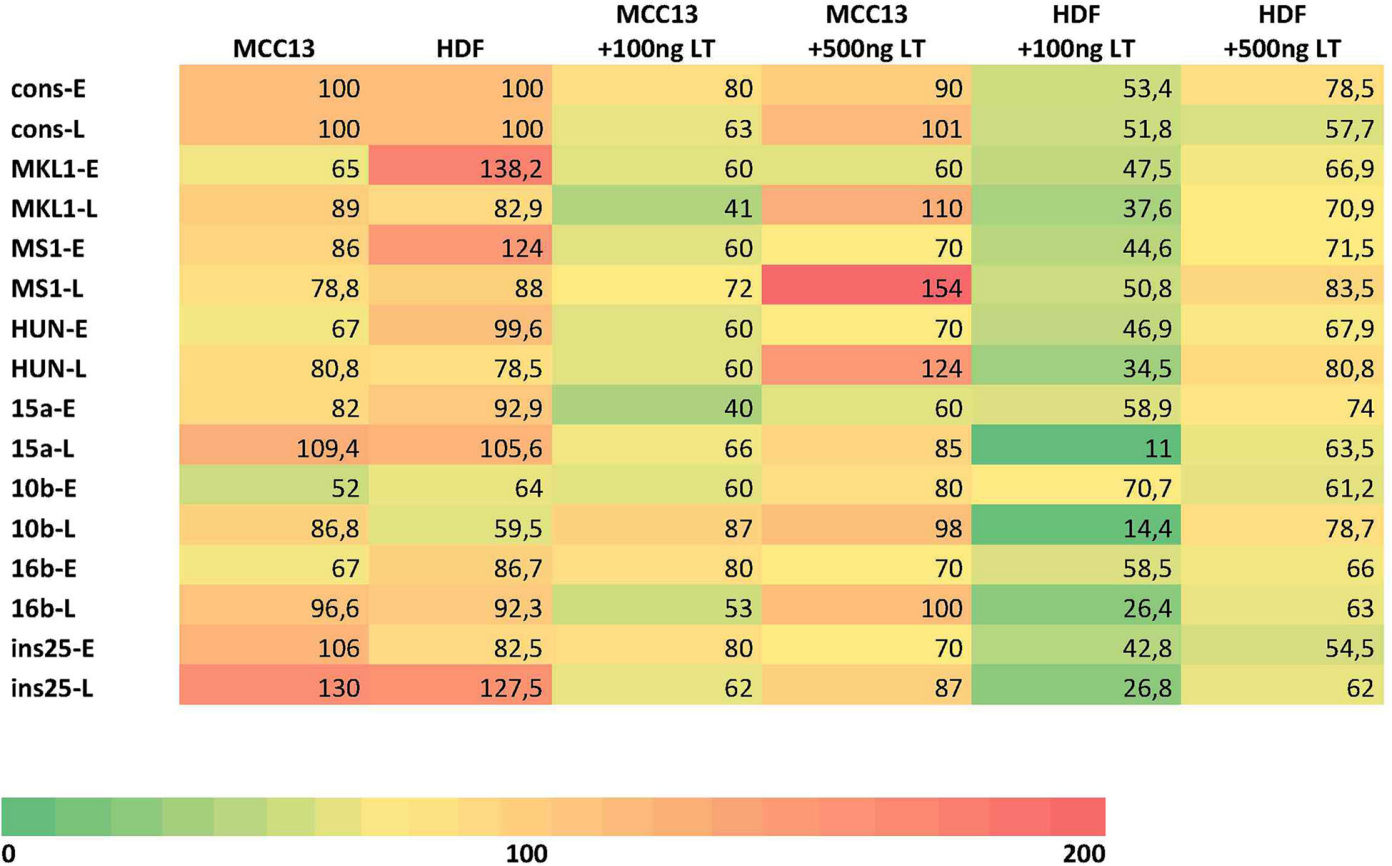
Heatmap showing the relative promoter activities of eight MCPyV NCCR variants in MCC13 and human dermal fibroblasts (HDF) in the absence and presence of large T antigen (LT). The activity of the consensus NCCR (cons) was arbitrary set as 100 and the activities of the other promoters were related to this

The MCPyV NCCR contains a plethora of putative transcription factor binding motifs (see Supplementary Fig. 3 and Supplementary Table S4), but the binding of the corresponding transcription factor has not been confirmed. Whether the mutations found in these sites in the NCCR variants we investigated abolished binding of the transcription factor, has not been tested. The 25 bp insertion generates putative binding motifs for the transcription factors FOXO3a, SRY, Elk-1 and p300, but their possible role in regulating the promoter activity remains to be investigated.

## Conclusions

Our study shows that the promoters of different MCPyV isolates possess unlike transcriptional activity, and that full-length LT and MCC-associated truncated LT have a distinct impact on the promoter. Whether these differences in promoter activity contribute to the replication and transformation properties of the virus remains to be determined.

## Supplementary information

**Additional file 1 Supplementary Fig. S1**. Early and late promoter activity of seven MCPyV NCCR variants in MCC13 and human dermal fibroblasts. **Supplementary Fig. S2**. Relative promoter activity of the immediately early cytomegalovirus and the consensus early MCPyV promoter in MCC13 and human dermal fibroblasts. **Supplementary Fig. S3**. Putative transcription factor binding sites in the consensus MCPyV NCCR. **Supplementary Table S1**. Detection of MCPyV in benign and diseased human tissues other than Merkel cell carcinoma. **Supplementary Table S2**. MCPyV isolated in sewage water and environmental surfaces. **Supplementary Table S3**. MCPyV NCCR variants and mutations compared to the consensus sequence. **Supplementary Table S4**. Putative transcription factor binding sites in the consensus MCPyV NCCR.

## Data Availability

The datasets used and/or analysed during the current study are available from the corresponding author on reasonable request.

## Abbreviations

HDF: Human dermal fibroblasts
LT: Large T antigen
MCC: Merkel cell carcinoma
MCPyV: Merkel cell polyomavirus
NCCR: Non-coding control region
sT: Small T antigen
tLT: Truncated large T antigen
