# Supplementary material for: Promoter activity of Merkel cell Polyomavirus variants in human dermal fibroblasts and a Merkel cell carcinoma cell line
Source: Virol J. 2020 Apr 19;17:54. doi: 10.1186/s12985-020-01317-x (PMC7168875; doi:10.1186/s12985-020-01317-x)
Supplement: Supplementary file 1 — Additional file 1 Supplementary Fig. S1. Early and late promoter activity of seven MCPyV NCCR variants in MCC13 and human dermal fibroblasts. Supplementary Fig. S2. Relative promoter activity of the immediately early cytomegalovirus and the consensus early MCPyV promoter in MCC13 and human dermal fibroblasts. Supplementary Fig. S3. Putative transcription factor binding sites in the consensus MCPyV NCCR. Supplementary Table S1. Detection of MCPyV in benign and diseased human tissues other than Merkel cell carcinoma. Supplementary Table S2. MCPyV isolated in sewage water and environmental surfaces. Supplementary Table S3. MCPyV NCCR variants and mutations compared to the consensus sequence. Supplementary Table S4. Putative transcription factor binding sites in the consensus MCPyV NCCR. [file 12985_2020_1317_MOESM1_ESM.pdf]

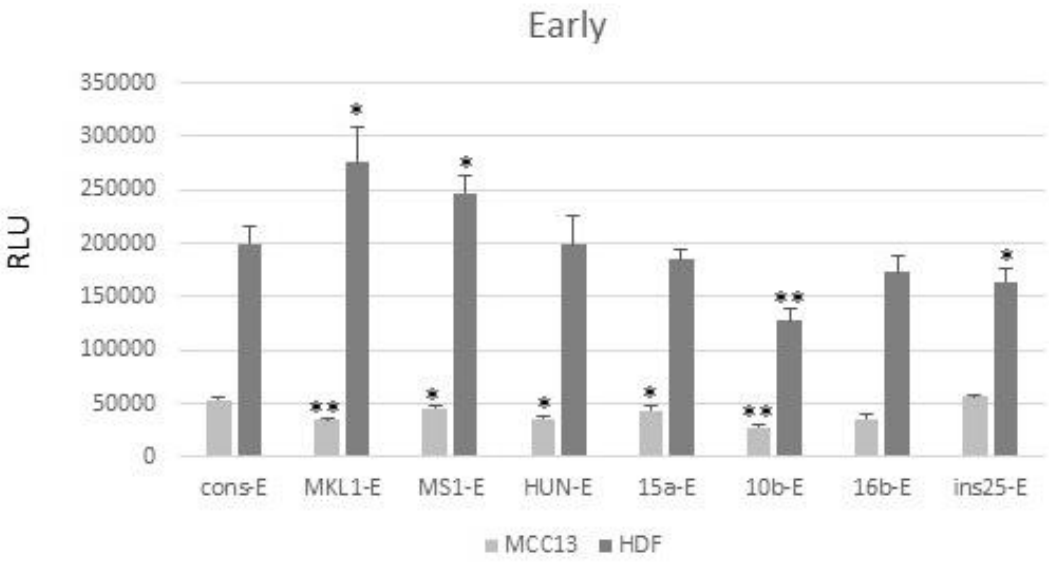

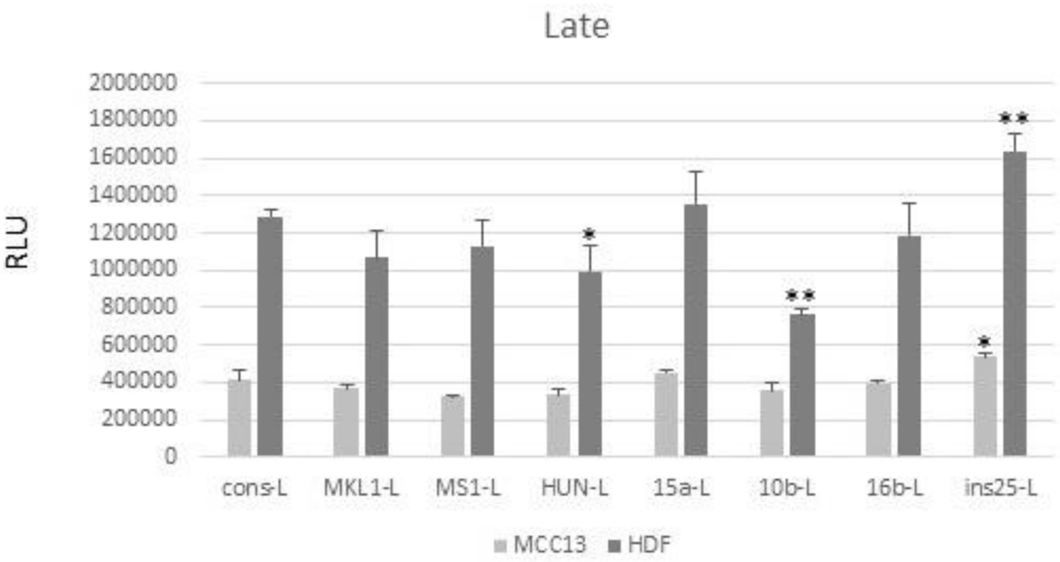

Supplementary Figure S1. Early and late promoter activity of seven MCPyV NCCR variants in MCC13 and human dermal fibroblasts. Promoter activities were determined by transient transfection studies with a luciferase reporter gene and are were corrected for the protein content in each sample. Promoter activity is expressed as relative luciferase activity (RLU).

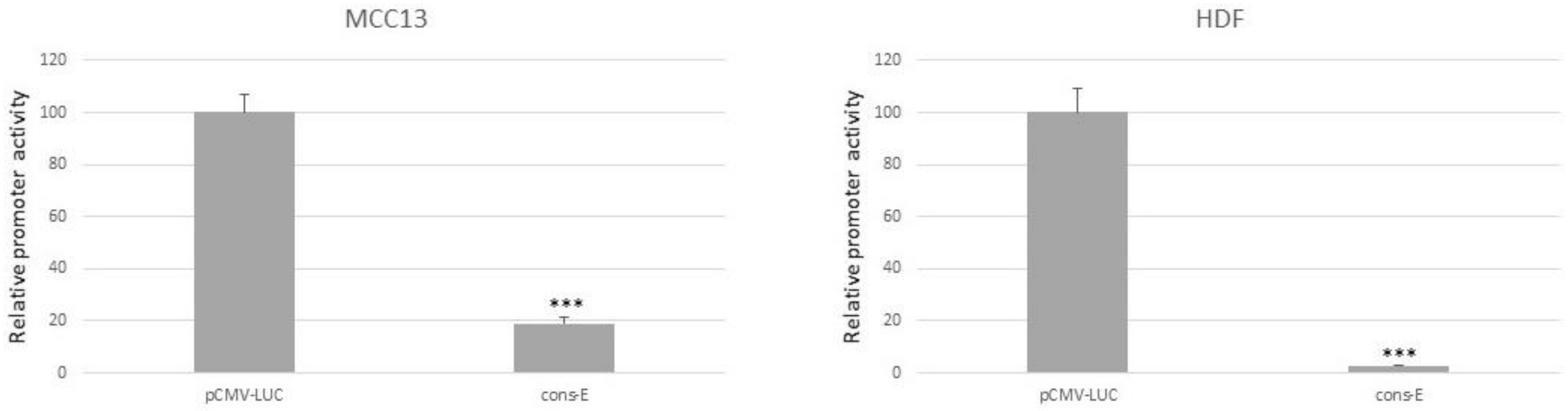

Supplementary Figure S2. Relative promoter activity of the immediately early cytomegalovirus and the consensus early MCPyV promoter in MCC13 and human dermal fibroblasts (HDF).

Supplementary Figure S3. Putative transcription factor binding sites in the consensus MCPyV NCCR. See Supplementary Table 4 for details.

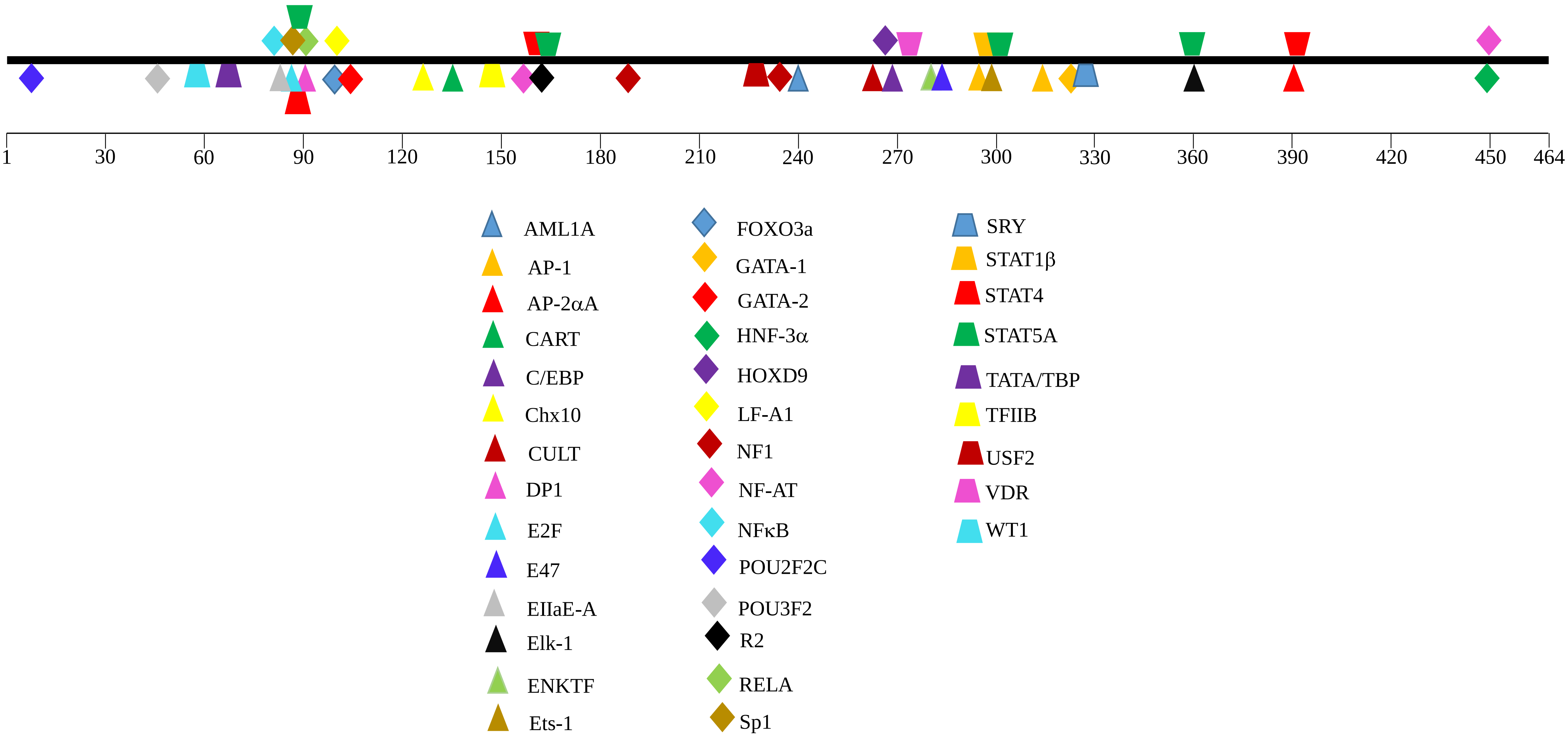

Supplementary Table S1. Detection of MCPyV in benign and diseased human tissues other than Merkel cell carcinoma.

System	Benign	Reference	Diseased	Reference
Circulatory system	Blood, plasma or serum from healthy donors	[1-8]	Cutaneous T- and B-cell lymphoma	[14-16]
	Sera from HIV-negative patients	[9]	Pseudolymphoma	[14]
	PBMC[a] healthy donors	[3]	Blood from a patient with Stevens-Johnson syndrome	[17]
	Whole blood from immunocompetent individuals	[10-12]	Blood from chronic lymphocytic leukemia patients	[2, 4, 8, 18-22]
			Blood renal transplant patients	[23, 24]
	Heart	[10, 13]		
			Inflammatory monocytes	[25]
			Blood MCC patients	[26]
			Blood from an acute myeloid leukemia patient	[27]
			Sera from HIV-positive patients	[9]
			PBMC from patients with Langerhans cell histiocytosis	[28]
			Non-Hodgkin lymphoma	[29, 30]
			Hodgkin lymphoma	[29]
			Whole blood from breast cancer patients	[31]
			Whole blood from immunocompromised patients	[12]
			Sera of AML[b] and ALL patients who underwent hematopoietic stem cell transplantation	[32]
			Blood polytransfused patients with hematological diseases	[8]
Digestive system	Saliva	[33-37]	Oral mucosa HIV-positive men	[26]
	Mouthwash healthy individuals	[38]	Colon cancer	[33, 39]

	Oral tissue/mucosa	[33, 35]	Liver from patients with cirrhosis	[10, 13, 33]
	Feces children	Li K[c]	Liver cancer	[10, 13, 33]
	Healthy colon tissue	[33, 39]	Anal cancer sample	[26]
	Stomach	[10, 13]	Anal mucosal samples HIV-positive men	[26]
	Liver	[10, 13, 33]	SCC[d] oral cavity	[33, 44, 45]
	Small intestine	[10, 13, 40]	SCC larynx	[46]
	Hemorrhoid	[40]	SCC esophagus	[33, 41, 42]
	Gall bladder	[13, 40]	Tumors of the salivary gland, including Warthin's tumor	[30, 47]
	Appendix	[40]	Head and neck cancer of throat, tongue, cheek, outer ear, larynx, mandibular	[48]
	Pancreas	[13]	Small cell cancer of the parotid	[49]
	Esophagus	[13, 33, 41, 42]	Oral tissue renal transplant patients	[34]
	Oral tissue	[34]	Oral and jaw tumors	[30]
	Salivary gland	[43]	Oral mucosa from patients with Langerhans cell histiocytosis	[28]
			Juvenile nasopharyngeal angiofibroma	[45]
			Feces thalassemic patients	[50]
			Feces acute myeloid leukemia patients	[50]
			Pleomorphic adenoma	[43]
			Warthin's tumor	[43]

			Salivary gland adenocarcinoma	[43]
Endocrine system	Adrenal gland<br><br>Thyroid gland	[13]<br><br>[13]	Pituitary from patients with Langerhans cell histiocytosis	[28]
Excretory System	Urine healthy<br><br>Urine bladder<br><br>Kidney	[23, 51-53]<br><br>[10, 13, 33]<br><br>[10, 13]	Urine renal transplant patients<br><br>Urine or tumor tissue of bladder cancer patients<br><br>Renal carcinoma<br><br>Urine patients with orthopedic disorder, acute surgical conditions or ear, nose, skin and dental disorder	[23, 24, 51-53]<br><br>[10, 13, 33, 54]<br><br>[33]<br><br>[54]
Integrumentary system	Normal skin/cutaneous swabs<br><br>Skin from patients with Verruca vulgaris<br><br>Eyebrow hairs<br><br>Inner root sheath cells of hair follicles	[13, 16, 40, 55-70]<br><br>[71-73]<br><br>[26, 61, 68, 69, 74-76]<br><br>[77]	Skin psoriasis patient<br><br>Actinic porokeratosis<br><br>Actinic keratosis<br><br>Skin lesions from chronic lymphocytic leukemia patients<br><br>Skin from patients with Langerhans cell histiocytosis<br><br>Skin patient with epidermodysplasia verruciformis<br><br>Verrucous papilloma of the skin<br><br>Cutaneous swabs forehead of HIV-positive patients<br><br>Skin patients with bullous pemphigoid<br><br>Skin patients with cutaneous drug side-effect<br><br>Skin from patients with seborrheic keratosis	[26, 33, 59, 66, 78]<br><br>[78, 79]<br><br>[73, 80-82]<br><br>[75]<br><br>[28]<br><br>[83, 84]<br><br>[85]<br><br>[60]<br><br>[59]<br><br>[59]<br><br>[71, 86, 87]

			Skin from patient with pruritic dermatosis	[88]
			Atopic dermatitis	[66]
			Palmoplantar pustulosis	[66]
			Skin from patients with mycosis fungoides	[15, 16, 59, 89]
			Fullicotropic mycosis fungoides (cutaneous T-cell lymphoma)	[90]
			Skin from T-cell mediated skin benign infiltrate	[66]
			Sézary syndrome	[15, 89]
			Fluid skin lesion Stevens-Johnson syndrome	[17]
			Kaposi's sarcoma	[40, 59, 78, 91, 92]
			Melanoma	[93]
			Cutaneous SCC	[28, 33, 38, 65, 67, 70, 72, 73, 76, 79, 82, 93-102]
			Bowen's disease	[71, 79, 94]
			Atypical fibroxanthoma	[82, 103]
			Porocarcinoma (cancer of the sweet glands)	[63, 65]
			Dermatofibrosarcoma protuberans	[79, 104]
			Keratoacanthoma	[79, 95]
			BCC[e]	[36, 70, 79, 81, 82, 93, 94, 105-107]
			Langerhans cell sarcoma	[108]

			Trichoblastoma<br><br>SCC of the skin<br><br>Spitz naevus<br><br>Non-melanoma skin cancer (including BCC, SCC, actinic keratosis, Bowen's disease)<br><br>Fanconi anemia-associated head and neck SCC	[105]<br><br>[107, 109]<br><br>[110]<br><br>[111]<br><br><br>[112]
Lymphatic system	Lymph nodes<br><br>Lymphoid tissue<br><br>Bone marrow<br><br>Spleen<br><br>Tonsils<br><br><br>Adenoid tissue	[29]<br><br>[113]<br><br>[13]<br><br>[13]<br><br>[1, 37, 114-118]<br><br>[116]	Tonsilar SCC<br><br>Epithelial thymic tumors	[114, 115]<br><br>[119]
Nervous system	Brain	[10]	CNS tumors	[30, 120]
Reproductive system	Prostate<br><br>Cervix<br><br>Ovary<br><br>Placenta<br><br>Testis<br><br>Vagina, cervix, vulvar skin, anus from immunodeficient patient with WILD syndrome	[13]<br><br>[121]<br><br>[10]<br><br>[122]<br><br>[13]<br><br>[26]	Prostatic secretion prostate cancer patients<br><br>Prostate cancer<br><br>Cervical SCC carcinoma<br><br>Cervical adenocarcinoma<br><br>Undifferentiated carcinomas of the cervix<br><br>Breast cancer	[123]<br><br>[33, 124]<br><br>[125, 126]<br><br>[125, 126]<br><br>[126]<br><br>[31, 127-130]

	(warts, immunodeficiency, lymphedema, anogenital dysplasia)		Benign breast disease<br><br>Testical cancer tissue (seminoma)<br><br>Penile mucosal HIV-positive men	[31]<br><br>[33]<br><br>[26]
Respiratory system	NPA[e]<br><br>lung	[1, 113, 131-135]<br><br>[10, 17, 33, 136]	NPA from patients with infectious diseases, hematology disorder, intensive care or cardiac or thoracic disease<br><br>NPA of renal transplant patients<br><br>NPA of cystic fibrosis patients<br><br>Non-small cell lung cancer<br><br>SCC of the lung<br><br>Adenocarcinoma<br><br>Large-cell neuroendocrine carcinoma<br><br>Pleomorphic carcinoma<br><br>Bronchoalveolar carcinoma<br><br>Lung cancer (not specified)<br><br>Small cell lung cancer<br><br>Extrapulmonary small cell carcinoma<br><br>Mesothelioma	[137]<br><br>[23]<br><br>[138]<br><br>[101, 139-144]<br><br>[100, 140, 145]<br><br>[100, 140-145]<br><br>[100]<br><br>[100]<br><br>[141]<br><br>[33]<br><br>[139, 146]<br><br>[147]<br><br>[148]
Skeletal system			Bone from patients with Langerhans cell histiocytosis<br><br>Ewing sarcoma	[28]<br><br>[102]

[a]Peripheral blood mononuclear cell

[b]Acute myeloid leukemia (AML) and acute lymphoblastic leukemia (ALL)

[c]Direct submission Li K

[d]Squamous cell carcinoma

[e]Basal cell carcinoma

[f]Nasopharyngeal aspirates

References

1. Kantola K, Sadeghi M, Lahtinen A, Koskenvuo M, Aaltonen LM, Mottonen M, Rahiala J, Saarinen-Pihkala U, Riikonen P, Jartti T *et al*: **Merkel cell polyomavirus DNA in tumor-free tonsillar tissues and upper respiratory tract samples: implications for respiratory transmission and latency**. *Journal of clinical virology : the official publication of the Pan American Society for Clinical Virology* 2009, **45**(4):292-295.
2. Pantulu ND, Pallasch CP, Kurz AK, Kassem A, Frenzel L, Sodenkamp S, Kvasnicka HM, Wendtner CM, Zur Hausen A: **Detection of a novel truncating Merkel cell polyomavirus large T antigen deletion in chronic lymphocytic leukemia cells**. *Blood* 2010, **116**(24):5280-5284.
3. Pancaldi C, Corazzari V, Maniero S, Mazzoni E, Comar M, Martini F, Tognon M: **Merkel cell polyomavirus DNA sequences in the buffy coats of healthy blood donors**. *Blood* 2011, **117**(26):7099-7101.
4. Imajoh M, Hashida Y, Taniguchi A, Kamioka M, Daibata M: **Novel human polyomaviruses, Merkel cell polyomavirus and human polyomavirus 9, in Japanese chronic lymphocytic leukemia cases**. *Journal of hematology & oncology* 2012, **5**:25.
5. Ngoi CN, Siqueira J, Li L, Deng X, Mugo P, Graham SM, Price MA, Sanders EJ, Delwart E: **The plasma virome of febrile adult Kenyans shows frequent parvovirus B19 infections and a novel arbovirus (Kadipiro virus)**. *J Gen Virol* 2016, **97**(12):3359-3367.
6. Lau P, Cordey S, Brito F, Tirefort D, Petty TJ, Turin L, Guichebaron A, Docquier M, Zdobnov EM, Waldvogel-Abramowski S *et al*: **Metagenomics analysis of red blood cell and fresh-frozen plasma units**. *Transfusion* 2017, **57**(7):1787-1800.
7. Mazzoni E, Rotondo JC, Marracino L, Selvatici R, Bononi I, Torreggiani E, Touze A, Martini F, Tognon MG: **Detection of Merkel Cell Polyomavirus DNA in Serum Samples of Healthy Blood Donors**. *Frontiers in oncology* 2017, **7**:294.
8. Comar M, Zanotta N, Del Savio R, Vascotto F, Calabrese N, Zorat F, Pozzato G: **No evidence of Polyomavirus and EBV infections in Italian patients with mixed cryoglobulinemia infected chronically with HCV**. *J Med Virol* 2014, **86**(4):666-671.
9. Fukumoto H, Sato Y, Hasegawa H, Katano H: **Frequent detection of Merkel cell polyomavirus DNA in sera of HIV-1-positive patients**. *Virol J* 2013, **10**:84.
10. Mancuso G, Antona J, Sirini C, Salvo M, Giacometti L, Olivero C, Trisolini E, Indellicato R, Boldorini R: **Frequent detection of Merkel cell polyomavirus DNA in tissues from 10 consecutive autopsies**. *J Gen Virol* 2017, **98**(6):1372-1376.
11. Shahzad N, Hussain I, Gilani US, Tayyeb A, Aslam MA, Khurshid M, Hassan U, Tasneem F, Umer M, Rashid N: **Merkel cell polyomavirus DNA sequences in the blood of healthy population of Pakistan**. *Future microbiology* 2019, **14**:599-608.

12. Jin HT, Park SJ, Choi EK, Kim YS: **The frequency of Merkel cell polyomavirus in whole blood from immunocompetent and immunosuppressed patients with kidney disease and healthy donors**. *Microbial pathogenesis* 2019, **131**:75-80.
13. Matsushita M, Kuwamoto S, Iwasaki T, Higaki-Mori H, Yashima S, Kato M, Murakami I, Horie Y, Kitamura Y, Hayashi K: **Detection of Merkel cell polyomavirus in the human tissues from 41 Japanese autopsy cases using polymerase chain reaction**. *Intervirology* 2013, **56**(1):1-5.
14. Andres C, Puchta U, Sander CA, Ruzicka T, Flaig MJ: **Prevalence of Merkel cell polyomavirus DNA in cutaneous lymphomas, pseudolymphomas, and inflammatory skin diseases**. *The American Journal of dermatopathology* 2010, **32**(6):593-598.
15. Kreuter A, Silling S, Dewan M, Stucker M, Wieland U: **Evaluation of 4 recently discovered human polyomaviruses in primary cutaneous B-cell and T-cell lymphoma**. *Archives of dermatology* 2011, **147**(12):1449-1451.
16. Du-Thanh A, Foulongne V, Guillot B, Dereure O: **Recently discovered human polyomaviruses in lesional and non-lesional skin of patients with primary cutaneous T-cell lymphomas**. *Journal of dermatological science* 2013, **71**(2):140-142.
17. Maximova N, Granzotto M, Kiren V, Zanon D, Comar M: **First description of Merkel Cell polyomavirus DNA detection in a patient with Stevens-Johnson syndrome**. *J Med Virol* 2013, **85**(5):918-923.
18. Teman CJ, Tripp SR, Perkins SL, Duncavage EJ: **Merkel cell polyomavirus (MCPyV) in chronic lymphocytic leukemia/small lymphocytic lymphoma**. *Leukemia research* 2011, **35**(5):689-692.
19. Haugg AM, Speel EJ, Pantulu ND, Pallasch C, Kurz AK, Kvasnicka HM, Cathomas G, Wendtner CM, zur Hausen A: **Fluorescence in situ hybridization confirms the presence of Merkel cell polyomavirus in chronic lymphocytic leukemia cells**. *Blood* 2011, **117**(21):5776-5777.
20. Comar M, Cuneo A, Maestri I, Melloni E, Pozzato G, Soffritti O, Secchiero P, Zauli G: **Merkel-cell polyomavirus (MCPyV) is rarely associated to B-chronic lymphocytic leukemia (1 out of 50) samples and occurs late in the natural history of the disease**. *Journal of clinical virology : the official publication of the Pan American Society for Clinical Virology* 2012, **55**(4):367-369.
21. Cimino PJ, Jr., Bahler DW, Duncavage EJ: **Detection of Merkel cell polyomavirus in chronic lymphocytic leukemia T-cells**. *Experimental and molecular pathology* 2013, **94**(1):40-44.
22. Trizuljak J, Srovnal J, Plevova K, Brychtova Y, Semerad L, Bakesova D, Letalova E, Benedikova A, Mayer J, Hajduch M *et al*: **Analysis of prognostic significance of merkel cell polyomavirus in chronic lymphocytic leukemia**. *Clinical lymphoma, myeloma & leukemia* 2015, **15**(7):439-442.
23. Bialasiewicz S, Rockett RJ, Barraclough KA, Leary D, Dudley KJ, Isbel NM, Sloots TP: **Detection of Recently Discovered Human Polyomaviruses in a Longitudinal Kidney Transplant Cohort**. *American journal of transplantation : official journal of the American Society of Transplantation and the American Society of Transplant Surgeons* 2016, **16**(9):2734-2740.
24. Wang Y, Strassl R, Helantera I, Aberle SW, Bond G, Hedman K, Weseslindtner L: **Multiplex analysis of Human Polyomavirus diversity in kidney transplant recipients with BK virus replication**. *Journal of clinical virology : the official publication of the Pan American Society for Clinical Virology* 2019, **120**:6-11.
25. Mertz KD, Junt T, Schmid M, Pfaltz M, Kempf W: **Inflammatory monocytes are a reservoir for Merkel cell polyomavirus**. *J Invest Dermatol* 2010, **130**(4):1146-1151.
26. Wieland U, Mauch C, Kreuter A, Krieg T, Pfister H: **Merkel cell polyomavirus DNA in persons without merkel cell carcinoma**. *Emerging infectious diseases* 2009, **15**(9):1496-1498.

27. Song Y, Gyarmati P: **Identification of Merkel Cell Polyomavirus from a Patient with Acute Myeloid Leukemia**. *Genome announcements* 2017, **5**(3).
28. Murakami I, Matsushita M, Iwasaki T, Kuwamoto S, Kato M, Horie Y, Hayashi K, Imamura T, Morimoto A, Imashuku S *et al*: **Merkel cell polyomavirus DNA sequences in peripheral blood and tissues from patients with Langerhans cell histiocytosis**. *Human pathology* 2014, **45**(1):119-126.
29. Toracchio S, Foyle A, Sroller V, Reed JA, Wu J, Kozinetz CA, Butel JS: **Lymphotropism of Merkel cell polyomavirus infection, Nova Scotia, Canada**. *Emerging infectious diseases* 2010, **16**(11):1702-1709.
30. Tanio S, Matsushita M, Kuwamoto S, Horie Y, Kodani I, Murakami I, Ryoke K, Hayashi K: **Low prevalence of Merkel cell polyomavirus with low viral loads in oral and maxillofacial tumours or tumour-like lesions from immunocompetent patients: Absence of Merkel cell polyomavirus-associated neoplasms**. *Molecular and clinical oncology* 2015, **3**(6):1301-1306.
31. Peng J, Wang T, Zhu H, Guo J, Li K, Yao Q, Lv Y, Zhang J, He C, Chen J *et al*: **Multiplex PCR/mass spectrometry screening of biological carcinogenic agents in human mammary tumors**. *Journal of clinical virology : the official publication of the Pan American Society for Clinical Virology* 2014, **61**(2):255-259.
32. Rahiala J, Koskenvuo M, Sadeghi M, Waris M, Vuorinen T, Lappalainen M, Saarinen-Pihkala U, Allander T, Soderlund-Venermo M, Hedman K *et al*: **Polyomaviruses BK, JC, KI, WU, MC, and TS in children with allogeneic hematopoietic stem cell transplantation**. *Pediatric transplantation* 2016, **20**(3):424-431.
33. Loyo M, Guerrero-Preston R, Brait M, Hoque MO, Chuang A, Kim MS, Sharma R, Liegeois NJ, Koch WM, Califano JA *et al*: **Quantitative detection of Merkel cell virus in human tissues and possible mode of transmission**. *Int J Cancer* 2010, **126**(12):2991-2996.
34. Baez CF, Guimaraes MA, Martins RA, Zalona AC, Cossatis JJ, Zalis MG, Cavalcanti SM, Varella RB: **Detection of Merkel cell polyomavirus in oral samples of renal transplant recipients without Merkel cell carcinoma**. *J Med Virol* 2013, **85**(11):2016-2019.
35. Freze Baez C, Cirauqui Diaz N, Baeta Cavalcanti SM, Brandao Varella R: **Genetic and structural analysis of Merkel cell polyomavirus large T antigen from diverse biological samples**. *Intervirology* 2014, **57**(6):331-336.
36. Baez CF, Diaz NC, Venceslau MT, Luz FB, Guimaraes MA, Zalis MG, Varella RB: **Phylogenetic and structural analysis of merkel cell polyomavirus VP1 in Brazilian samples**. *Virus research* 2016, **221**:1-7.
37. Kourieh A, Combes JD, Tommasino M, Dalstein V, Clifford GM, Lacau St Guily J, Clavel C, Franceschi S, Gheit T, For The Split Study G: **Prevalence and risk factors of human polyomavirus infections in non-malignant tonsils and gargles: the SPLIT study**. *J Gen Virol* 2018, **99**(12):1686-1698.
38. Dworkin AM, Tseng SY, Allain DC, Iwenofu OH, Peters SB, Toland AE: **Merkel cell polyomavirus in cutaneous squamous cell carcinoma of immunocompetent individuals**. *J Invest Dermatol* 2009, **129**(12):2868-2874.
39. Campello C, Comar M, D'Agaro P, Minicozzi A, Rodella L, Poli A: **A molecular case-control study of the Merkel cell polyomavirus in colon cancer**. *J Med Virol* 2011, **83**(4):721-724.
40. Feng H, Shuda M, Chang Y, Moore PS: **Clonal integration of a polyomavirus in human Merkel cell carcinoma**. *Science (New York, NY)* 2008, **319**(5866):1096-1100.
41. Yahyapour Y, Sadeghi F, Alizadeh A, Rajabnia R, Siadati S: **Detection of Merkel Cell Polyomavirus and Human Papillomavirus in Esophageal Squamous Cell Carcinomas and Non-Cancerous Esophageal Samples in Northern Iran**. *Pathology oncology research : POR* 2016, **22**(4):667-672.

42. Yahyapour Y, Rahmani R, Alipour M, Alizadeh A, Khademian A, Sadeghi F: **Prevalence and association of human papillomavirus, Epstein-Barr virus and Merkel Cell polyomavirus with neoplastic esophageal lesions in northern Iran**. *Caspian journal of internal medicine* 2018, **9**(4):353-360.
43. Chen AA, Gheit T, Stellin M, Lupato V, Spinato G, Fuson R, Menegaldo A, McKay-Chopin S, Dal Cin E, Tirelli G *et al*: **Oncogenic DNA viruses found in salivary gland tumors**. *Oral Oncol* 2017, **75**:106-110.
44. Hamiter M, Asarkar A, Rogers D, Moore-Medlin T, McClure G, Ma X, Vanchiere J, Nathan CO: **A pilot study of Merkel cell polyomavirus in squamous cell carcinoma of the tongue**. *Oral Oncol* 2017, **74**:111-114.
45. Dickinson A, Xu M, Silen S, Wang Y, Fu Y, Sadeghi M, Toppinen M, Carpen T, Hedman K, Makitie A *et al*: **Newly detected DNA viruses in juvenile nasopharyngeal angiofibroma (JNA) and oral and oropharyngeal squamous cell carcinoma (OSCC/OPSCC)**. *European archives of oto-rhino-laryngology : official journal of the European Federation of Oto-Rhino-Laryngological Societies (EUFOS) : affiliated with the German Society for Oto-Rhino-Laryngology - Head and Neck Surgery* 2019, **276**(2):613-617.
46. Vazquez-Guillen JM, Palacios-Saucedo GC, Rivera-Morales LG, Alonzo-Morado MV, Burciaga-Bernal SB, Montufar-Martinez M, Ortiz-Lopez R, Gonzalez-Villasana V, Martinez-Torres AC, Serna-Hernandez JC *et al*: **Infection and coinfection by human papillomavirus, Epstein-Barr virus and Merkel cell polyomavirus in patients with squamous cell carcinoma of the larynx: a retrospective study**. *PeerJ* 2018, **6**:e5834.
47. Ramqvist T, Ursu RG, Haeggblom L, Mirzaie L, Gahm C, Hammarstedt-Nordenvall L, Dalianis T, Nasman A: **Human Polyomaviruses Are Not Frequently Present in Cancer of the Salivary Glands**. *Anticancer research* 2018, **38**(5):2871-2874.
48. Mohebbi E, Noormohamadi Z, Sadeghi-Rad H, Sadeghi F, Yahyapour Y, Vaziri F, Rahimi A, Rahimi Jamnani F, Mehrabi S, Siadat SD *et al*: **Low viral load of Merkel cell polyomavirus in Iranian patients with head and neck squamous cell carcinoma: Is it clinically important?** *J Med Virol* 2018, **90**(2):344-350.
49. Fisher CA, Harms PW, McHugh JB, Edwards PC, Siddiqui J, Palanisamy N, Bichakjian CK, Benavides E, Danciu TE: **Small cell carcinoma in the parotid harboring Merkel cell polyomavirus**. *Oral surgery, oral medicine, oral pathology and oral radiology* 2014, **118**(6):703-712.
50. Prezioso C, Ciotti M, Obregon F, Ambroselli D, Rodio DM, Cudillo L, Gaziev J, Mele A, Nardi A, Favalli C *et al*: **Polyomaviruses shedding in stool of patients with hematological disorders: detection analysis and study of the non-coding control region's genetic variability**. *Medical microbiology and immunology* 2019.
51. Husseiny MI, Anastasi B, Singer J, Lacey SF: **A comparative study of Merkel cell, BK and JC polyomavirus infections in renal transplant recipients and healthy subjects**. *Journal of clinical virology : the official publication of the Pan American Society for Clinical Virology* 2010, **49**(2):137-140.
52. Signorini L, Belingheri M, Ambrogi F, Pagani E, Binda S, Ticozzi R, Ferraresso M, Ghio L, Giacon B, Ferrante P *et al*: **High frequency of Merkel cell polyomavirus DNA in the urine of kidney transplant recipients and healthy controls**. *Journal of clinical virology : the official publication of the Pan American Society for Clinical Virology* 2014, **61**(4):565-570.
53. Torres C, Barrios ME, Cammarata RV, Cisterna DM, Estrada T, Martini Novas S, Cahn P, Blanco Fernandez MD, Mbayed VA: **High diversity of human polyomaviruses in environmental and clinical samples in Argentina: Detection of JC, BK, Merkel-cell, Malawi, and human 6 and 7 polyomaviruses**. *The Science of the total environment* 2016, **542**(Pt A):192-202.
54. Polesel J, Gheit T, Talamini R, Shahzad N, Lenardon O, Sylla B, La Vecchia C, Serraino D, Tommasino M, Franceschi S: **Urinary human polyomavirus and papillomavirus infection and bladder cancer risk**. *Br J Cancer* 2012, **106**(1):222-226.

55. Schowalter RM, Pastrana DV, Pumphrey KA, Moyer AL, Buck CB: **Merkel cell polyomavirus and two previously unknown polyomaviruses are chronically shed from human skin**. *Cell Host Microbe* 2010, **7**(6):509-515.
56. Foulongne V, Sauvage V, Hebert C, Dereure O, Cheval J, Gouilh MA, Pariente K, Segondy M, Burguiere A, Manuguerra JC *et al*: **Human skin microbiota: high diversity of DNA viruses identified on the human skin by high throughput sequencing**. *PLoS One* 2012, **7**(6):e38499.
57. Martel-Jantin C, Filippone C, Tortevoye P, Afonso PV, Betsem E, Descorps-Declere S, Nicol JT, Touze A, Coursaget P, Crouzat M *et al*: **Molecular epidemiology of merkel cell polyomavirus: evidence for geographically related variant genotypes**. *J Clin Microbiol* 2014, **52**(5):1687-1690.
58. Hashida Y, Higuchi T, Matsui K, Shibata Y, Nakajima K, Sano S, Daibata M: **Genetic Variability of the Noncoding Control Region of Cutaneous Merkel Cell Polyomavirus: Identification of Geographically Related Genotypes**. *J Infect Dis* 2018, **217**(10):1601-1611.
59. Foulongne V, Kluger N, Dereure O, Mercier G, Moles JP, Guillot B, Segondy M: **Merkel cell polyomavirus in cutaneous swabs**. *Emerging infectious diseases* 2010, **16**(4):685-687.
60. Wieland U, Silling S, Scola N, Potthoff A, Gambichler T, Brockmeyer NH, Pfister H, Kreuter A: **Merkel cell polyomavirus infection in HIV-positive men**. *Archives of dermatology* 2011, **147**(4):401-406.
61. Hampras SS, Giuliano AR, Lin HY, Fisher KJ, Abrahamsen ME, McKay-Chopin S, Gheit T, Tommasino M, Rollison DE: **Natural history of polyomaviruses in men: the HPV infection in men (HIM) study**. *J Infect Dis* 2015, **211**(9):1437-1446.
62. Hashida Y, Kamioka M, Tanaka M, Hosokawa S, Murakami M, Nakajima K, Kikuchi H, Fujieda M, Sano S, Daibata M: **Ecology of Merkel Cell Polyomavirus in Healthy Skin Among Individuals in an Asian Cohort**. *J Infect Dis* 2016, **213**(11):1708-1716.
63. Urso C, Pierucci F, Sollai M, Arvia R, Massi D, Zakrzewska K: **Detection of Merkel cell polyomavirus and human papillomavirus DNA in porocarcinoma**. *Journal of clinical virology : the official publication of the Pan American Society for Clinical Virology* 2016, **78**:71-73.
64. Hashida Y, Nakajima K, Nakajima H, Shiga T, Tanaka M, Murakami M, Matsuzaki S, Naganuma S, Kuroda N, Seki Y *et al*: **High load of Merkel cell polyomavirus DNA detected in the normal skin of Japanese patients with Merkel cell carcinoma**. *Journal of clinical virology : the official publication of the Pan American Society for Clinical Virology* 2016, **82**:101-107.
65. Arvia R, Sollai M, Pierucci F, Urso C, Massi D, Zakrzewska K: **Droplet digital PCR (ddPCR) vs quantitative real-time PCR (qPCR) approach for detection and quantification of Merkel cell polyomavirus (MCPyV) DNA in formalin fixed paraffin embedded (FFPE) cutaneous biopsies**. *Journal of virological methods* 2017, **246**:15-20.
66. Hashida Y, Higuchi T, Tanaka M, Shibata Y, Nakajima K, Sano S, Daibata M: **Prevalence and Viral Loads of Cutaneous Human Polyomaviruses in the Skin of Patients With Chronic Inflammatory Skin Diseases**. *J Infect Dis* 2019, **219**(10):1564-1573.
67. Wang Y, Keinonen A, Koskenmies S, Pitkanen S, Fyhrquist N, Sadeghi M, Makisalo H, Soderlund-Venermo M, Hedman K: **Occurrence of newly discovered human polyomaviruses in skin of liver transplant recipients and their relation with squamous cell carcinoma in situ and actinic keratosis - a single-center cohort study**. *Transplant international : official journal of the European Society for Organ Transplantation* 2019, **32**(5):516-522.
68. Amorrortu RP, Fenske NA, Cherpelis BS, Vijayan L, Zhao Y, Balliu J, Messina JL, Sondak VK, Giuliano AR, Gheit T *et al*: **Viruses in Skin Cancer (VIRUSCAN): Study design and baseline characteristics of a prospective clinic-based cohort study**. *Cancer epidemiology, biomarkers & prevention : a publication of the American Association for Cancer Research, cosponsored by the American Society of Preventive Oncology* 2019.

69. Rollison DE, Schell MJ, Fenske NA, Cherpelis B, Messina JL, Giuliano AR, Epling-Burnette PK, Hampras SS, Amorrortu RP, Balliu J *et al*: **Cutaneous Viral Infections Across 2 Anatomic Sites Among a Cohort of Patients Undergoing Skin Cancer Screening**. *J Infect Dis* 2019, **219**(5):711-722.
70. Mertz KD, Paasinen A, Arnold A, Baumann M, Offner F, Willi N, Cathomas G: **Merkel cell polyomavirus large T antigen is detected in rare cases of nonmelanoma skin cancer**. *Journal of cutaneous pathology* 2013, **40**(6):543-549.
71. Mertz KD, Pfaltz M, Junt T, Schmid M, Fernandez Figueras MT, Pfaltz K, Barghorn A, Kempf W: **Merkel cell polyomavirus is present in common warts and carcinoma in situ of the skin**. *Human pathology* 2010, **41**(10):1369-1379.
72. Falchook GS, Rady P, Konopinski JC, Busaidy N, Hess K, Hymes S, Nguyen HP, Prieto VG, Bustinza-Linares E, Lin Q *et al*: **Merkel cell polyomavirus and human papilloma virus in proliferative skin lesions arising in patients treated with BRAF inhibitors**. *Archives of dermatological research* 2016, **308**(5):357-365.
73. Purdie KJ, Proby CM, Rizvi H, Griffin H, Doorbar J, Sommerlad M, Feltkamp MC, der Meijden EV, Inman GJ, South AP *et al*: **The Role of Human Papillomaviruses and Polyomaviruses in BRAF-Inhibitor Induced Cutaneous Squamous Cell Carcinoma and Benign Squamoproliferative Lesions**. *Frontiers in microbiology* 2018, **9**:1806.
74. Bellaud G, Gheit T, Pugin A, Pretet JL, Tommasino M, Mougin C, Aubin F: **Prevalence of human polyomavirus DNA in eyebrow hairs plucked from patients with psoriasis treated with TNF inhibitors**. *Journal of the European Academy of Dermatology and Venereology : JEADV* 2015, **29**(5):1019-1021.
75. Peretti A, Borgogna C, Rossi D, De Paoli L, Bawadekar M, Zavattaro E, Boldorini R, De Andrea M, Gaidano G, Gariglio M: **Analysis of human beta-papillomavirus and Merkel cell polyomavirus infection in skin lesions and eyebrow hair bulbs from a cohort of patients with chronic lymphocytic leukaemia**. *Br J Dermatol* 2014, **171**(6):1525-1528.
76. Hampras SS, Michel A, Schmitt M, Waterboer T, Kranz L, Gheit T, Fisher K, Sondak VK, Messina J, Fenske N *et al*: **Merkel cell polyomavirus (MCV) T-antigen seroreactivity, MCV DNA in eyebrow hairs, and squamous cell carcinoma**. *Infectious agents and cancer* 2015, **10**:35.
77. Nemeth K, Gorog A, Mezey E, Pinter D, Kuroli E, Harsing J, Kovacs L, Fischer M, Rady P, Sillo P *et al*: **Image Correspondence: Cover Image: Detection of hair follicleassociated Merkel cell polyomavirus in an immunocompromised host with follicular spicules and alopecia**. *Br J Dermatol* 2016, **175**(6):1409.
78. Foulongne V, Dereure O, Kluger N, Moles JP, Guillot B, Segondy M: **Merkel cell polyomavirus DNA detection in lesional and nonlesional skin from patients with Merkel cell carcinoma or other skin diseases**. *Br J Dermatol* 2010, **162**(1):59-63.
79. Bellott TR, Baez CF, Almeida SG, Venceslau MT, Zalis MG, Guimaraes MA, Rochael MC, Luz FB, Varella RB, Almeida JR: **Molecular prevalence of Merkel cell polyomavirus in nonmelanoma skin cancer in a Brazilian population**. *Clinical and experimental dermatology* 2017, **42**(4):390-394.
80. Ridd K, Yu S, Bastian BC: **The presence of polyomavirus in non-melanoma skin cancer in organ transplant recipients is rare**. *J Invest Dermatol* 2009, **129**(1):250-252.
81. Ota S, Ishikawa S, Takazawa Y, Goto A, Fujii T, Ohashi K, Fukayama M: **Quantitative analysis of viral load per haploid genome revealed the different biological features of Merkel cell polyomavirus infection in skin tumor**. *PLoS One* 2012, **7**(6):e39954.
82. Scola N, Wieland U, Silling S, Altmeyer P, Stucker M, Kreuter A: **Prevalence of human polyomaviruses in common and rare types of non-Merkel cell carcinoma skin cancer**. *Br J Dermatol* 2012, **167**(6):1315-1320.

83. Mertz KD, Schmid M, Burger B, Itin P, Palmedo G, Scharer L, Kutzner H, Fernandez Figueras MT, Cribier B, Pfaltz M *et al*: **Detection of Merkel cell polyomavirus in epidermodysplasia-verruciformis-associated skin neoplasms**. *Dermatology (Basel, Switzerland)* 2011, **222**(1):87-92.
84. Kwon EK, Halvorson CR, Rady P, Tyring S, Nguyen HP, Kao GF, Gaspari AA: **Merkel cell polyomavirus detection in a patient with familial epidermodysplasia verruciformis**. *Pediatric dermatology* 2013, **30**(4):505-507.
85. Frouin E, Guillot B, Larrieux M, Tempier A, Boulle N, Foulongne V, Girard C, Costes V, Solassol J: **Cutaneous epithelial tumors induced by vemurafenib involve the MAPK and Pi3KCA pathways but not HPV nor HPyV viral infection**. *PLoS One* 2014, **9**(10):e110478.
86. Andres C, Belloni B, Puchta U, Sander CA, Flaig MJ: **Prevalence of MCPyV in Merkel cell carcinoma and non-MCC tumors**. *Journal of cutaneous pathology* 2010, **37**(1):28-34.
87. Hillen LM, Rennspiess D, Speel EJ, Haugg AM, Winnepenninckx V, Zur Hausen A: **Detection of Merkel Cell Polyomavirus in Seborrheic Keratosis**. *Frontiers in microbiology* 2017, **8**:2648.
88. Nguyen KD, Lee EE, Yue Y, Stork J, Pock L, North JP, Vandergriff T, Cockerell C, Hosler GA, Pastrana DV *et al*: **Human polyomavirus 6 and 7 are associated with pruritic and dyskeratotic dermatoses**. *J Am Acad Dermatol* 2017, **76**(5):932-940.e933.
89. Du-Thanh A, Dereure O, Guillot B, Foulongne V: **Merkel cell polyomavirus: its putative involvement in a particular subset of cutaneous lymphoma with possibly unfavorable outcome**. *Journal of clinical virology : the official publication of the Pan American Society for Clinical Virology* 2014, **61**(1):161-165.
90. Gormley RH, Kim EJ, Rook AH, Kovarik CL, Sun A, Rady P, Tyring SK: **Merkel cell polyomavirus in low levels in folliculotropic mycosis fungoides represents a passenger, not a driver**. *International journal of dermatology* 2015, **54**(5):e182-183.
91. Katano H, Ito H, Suzuki Y, Nakamura T, Sato Y, Tsuji T, Matsuo K, Nakagawa H, Sata T: **Detection of Merkel cell polyomavirus in Merkel cell carcinoma and Kaposi's sarcoma**. *J Med Virol* 2009, **81**(11):1951-1958.
92. Du-Thanh A, Guillot B, Dereure O, Foulongne V: **Detection of Merkel cell and other human polyomavirus DNA in lesional and nonlesional skin from patients with Kaposi sarcoma**. *Br J Dermatol* 2015, **173**(4):1063-1065.
93. Imajoh M, Hashida Y, Nakajima H, Sano S, Daibata M: **Prevalence and viral DNA loads of three novel human polyomaviruses in skin cancers from Japanese patients**. *The Journal of dermatology* 2013, **40**(8):657-660.
94. Kassem A, Technau K, Kurz AK, Pantulu D, Loning M, Kayser G, Stickeler E, Weyers W, Diaz C, Werner M *et al*: **Merkel cell polyomavirus sequences are frequently detected in nonmelanoma skin cancer of immunosuppressed patients**. *Int J Cancer* 2009, **125**(2):356-361.
95. Wieland U, Scola N, Stolte B, Stucker M, Silling S, Kreuter A: **No evidence for a causal role of Merkel cell polyomavirus in keratoacanthoma**. *J Am Acad Dermatol* 2012, **67**(1):41-46.
96. Kaibuchi-Noda K, Yokota K, Matsumoto T, Sawada M, Sakakibara A, Kono M, Tomita Y, Watanabe D, Fukumoto H, Katano H *et al*: **Detection of Merkel cell polyomavirus in cutaneous squamous cell carcinoma before occurrence of Merkel cell carcinoma**. *J Am Acad Dermatol* 2011, **65**(5):e152-154.
97. Rollison DE, Giuliano AR, Messina JL, Fenske NA, Cherpelis BS, Sondak VK, Roetzheim RG, Iannacone MR, Michael KM, Gheit T *et al*: **Case-control study of Merkel cell polyomavirus infection and cutaneous squamous cell carcinoma**. *Cancer epidemiology, biomarkers & prevention : a publication of the American Association for Cancer Research, cosponsored by the American Society of Preventive Oncology* 2012, **21**(1):74-81.

98. Mitteldorf C, Mertz KD, Fernandez-Figueras MT, Schmid M, Tronnier M, Kempf W: **Detection of Merkel cell polyomavirus and human papillomaviruses in Merkel cell carcinoma combined with squamous cell carcinoma in immunocompetent European patients**. *The American Journal of dermatopathology* 2012, **34**(5):506-510.
99. Falchook GS, Rady P, Hymes S, Nguyen HP, Tyring SK, Prieto VG, Hong DS, Kurzrock R: **Merkel cell polyomavirus and HPV-17 associated with cutaneous squamous cell carcinoma arising in a patient with melanoma treated with the BRAF inhibitor dabrafenib**. *JAMA dermatology* 2013, **149**(3):322-326.
100. Hashida Y, Imajoh M, Nemoto Y, Kamioka M, Taniguchi A, Taguchi T, Kume M, Orihashi K, Daibata M: **Detection of Merkel cell polyomavirus with a tumour-specific signature in non-small cell lung cancer**. *Br J Cancer* 2013, **108**(3):629-637.
101. Hashida Y, Imajoh M, Kamioka M, Taniguchi A, Kuroda N, Hayashi K, Nakajima H, Sano S, Daibata M: **Phylogenetic analysis of Merkel cell polyomavirus based on full-length LT and VP1 gene sequences derived from neoplastic tumours in Japanese patients**. *J Gen Virol* 2014, **95**(Pt 1):135-141.
102. Jung HS, Choi YL, Choi JS, Roh JH, Pyon JK, Woo KJ, Lee EH, Jang KT, Han J, Park CS *et al*: **Detection of Merkel cell polyomavirus in Merkel cell carcinomas and small cell carcinomas by PCR and immunohistochemistry**. *Histology and histopathology* 2011, **26**(10):1231-1241.
103. Andres C, Puchta U, Flaig MJ: **Detection of Merkel cell polyomavirus DNA in atypical fibroxanthoma in correlation to clinical features**. *The American Journal of dermatopathology* 2010, **32**(8):799-803.
104. Aung PP, Rady PL, Nagarajan P, Ivan D, Tetzlaff MT, Curry JL, Tyring SK, Prieto VG, Torres-Cabala CA: **Detection of Merkel Cell Polyoma Virus and Beta Human Papillomavirus in Multiple Eccrine Poromas in a Patient With Acute Leukemia Treated With Stem Cell Transplant**. *The American Journal of dermatopathology* 2017, **39**(6):489-491.
105. Kassem A, Pantulu D, Technau K, Kurz AK, Diaz C, Horster S, Nashan D, Weyers W, Zur Hausen A: **Merkel cell polyomavirus in naevoid basal cell carcinoma syndrome-associated basal cell carcinomas and sporadic trichoblastomas**. *Journal of dermatological science* 2010, **59**(2):140-142.
106. Iwasaki T, Kodama H, Matsushita M, Kuroda N, Yamasaki Y, Murakami I, Yamamoto O, Hayashi K: **Merkel cell polyomavirus infection in both components of a combined Merkel cell carcinoma and basal cell carcinoma with ductal differentiation; each component had a similar but different novel Merkel cell polyomavirus large T antigen truncating mutation**. *Human pathology* 2013, **44**(3):442-447.
107. Neto CF, Oliveira WRP, Costa PVA, Cardoso MK, Barreto PG, Romano CM, Urbano PR: **The first observation of the association of Merkel cell polyomavirus and Merkel cell carcinoma in Brazil**. *International journal of dermatology* 2019, **58**(6):703-706.
108. Murakami I, Matsushita M, Iwasaki T, Kuwamoto S, Kato M, Horie Y, Hayashi K, Gogusev J, Jaubert F, Nakamoto S *et al*: **High viral load of Merkel cell polyomavirus DNA sequences in Langerhans cell sarcoma tissues**. *Infectious agents and cancer* 2014, **9**:15.
109. Schrama D, Groesser L, Ugurel S, Hafner C, Pastrana DV, Buck CB, Cerroni L, Theiler A, Becker JC: **Presence of human polyomavirus 6 in mutation-specific BRAF inhibitor-induced epithelial proliferations**. *JAMA dermatology* 2014, **150**(11):1180-1186.
110. Haeggblom L, Franzen J, Nasman A: **Human polyomavirus DNA detection in keratoacanthoma and Spitz naevus: no evidence for a causal role**. *Journal of clinical pathology* 2017, **70**(5):451-453.
111. Baez CF, Goncalves MTV, da Rocha WM, Magalhaes de Souza L, Savassi-Ribas F, de Oliveira Almeida NK, Delbue S, Guimaraes M, Cavalcanti SMB, Luz FB *et al*: **Investigation of three oncogenic epitheliotropic viruses shows human papillomavirus in association with non-melanoma skin cancer**.

European journal of clinical microbiology & infectious diseases : official publication of the European Society of Clinical Microbiology 2019, **38**(6):1129-1133.

112. Toptan T, Brusadelli MG, Turpin B, Witte DP, Surralles J, Velleuer E, Schramm M, Dietrich R, Brakenhoff RH, Moore PS *et al*: **Limited detection of human polyomaviruses in Fanconi anemia related squamous cell carcinoma**. *PLoS One* 2018, **13**(12):e0209235.
113. Sharp CP, Norja P, Anthony I, Bell JE, Simmonds P: **Reactivation and mutation of newly discovered WU, KI, and Merkel cell carcinoma polyomaviruses in immunosuppressed individuals**. *J Infect Dis* 2009, **199**(3):398-404.
114. Salakova M, Koslabova E, Vojtechova Z, Tachezy R, Sroller V: **Detection of human polyomaviruses MCPyV, HPyV6, and HPyV7 in malignant and non-malignant tonsillar tissues**. *J Med Virol* 2016, **88**(4):695-702.
115. Herberhold S, Hellmich M, Panning M, Bartok E, Silling S, Akgul B, Wieland U: **Human polyomavirus and human papillomavirus prevalence and viral load in non-malignant tonsillar tissue and tonsillar carcinoma**. *Medical microbiology and immunology* 2017, **206**(2):93-103.
116. Cason C, Monasta L, Zanotta N, Campisciano G, Maestri I, Tommasino M, Pawlita M, Villani S, Comar M, Delbue S: **Antibody response to polyomavirus primary infection: high seroprevalence of Merkel cell polyomavirus and lymphoid tissue involvement**. *J Neurovirol* 2018, **24**(3):314-322.
117. Peng J, Li K, Zhang C, Jin Q: **MW polyomavirus and STL polyomavirus present in tonsillar tissues from children with chronic tonsillar disease**. *Clinical microbiology and infection : the official publication of the European Society of Clinical Microbiology and Infectious Diseases* 2016, **22**(1):97.e91-97.e93.
118. Sadeghi M, Wang Y, Ramqvist T, Aaltonen LM, Pyoria L, Toppinen M, Soderlund-Venermo M, Hedman K: **Multiplex detection in tonsillar tissue of all known human polyomaviruses**. *BMC infectious diseases* 2017, **17**(1):409.
119. Chteinberg E, Klufah F, Rennspiess D, Mannheims MF, Abdul-Hamid MA, Losen M, Keijzers M, De Baets MH, Kurz AK, Zur Hausen A: **Low prevalence of Merkel cell polyomavirus in human epithelial thymic tumors**. *Thoracic cancer* 2019, **10**(3):445-451.
120. Sadeghi F, Salehi-Vaziri M, Alizadeh A, Ghodsi SM, Bokharaei-Salim F, Fateh A, Monavari SH, Keyvani H: **Detection of Merkel cell polyomavirus large T-antigen sequences in human central nervous system tumors**. *J Med Virol* 2015, **87**(7):1241-1247.
121. Kolia-Diafouka P, Foulongne V, Boulle N, Ngou J, Kelly H, Sawadogo B, Delany-Moretlwe S, Mayaud P, Segondy M: **Detection of four human polyomaviruses (MCPyV, HPyV6, HPyV7 and TSPyV) in cervical specimens from HIV-infected and HIV-uninfected women**. *Sexually transmitted infections* 2016, **92**(7):492-494.
122. Sadeghi M, Riipinen A, Vaisanen E, Chen T, Kantola K, Surcel HM, Karikoski R, Taskinen H, Soderlund-Venermo M, Hedman K: **Newly discovered KI, WU, and Merkel cell polyomaviruses: no evidence of mother-to-fetus transmission**. *Virol J* 2010, **7**:251.
123. Smelov V, Bzhalava D, Arroyo Muhr LS, Eklund C, Komyakov B, Gorelov A, Dillner J, Hultin E: **Detection of DNA viruses in prostate cancer**. *Scientific reports* 2016, **6**:25235.
124. Khan G, Philip PS, Naase M, Al Zarouni KM: **No evidence for the involvement of XMRV or MCV in the pathogenesis of breast cancer**. *Br J Cancer* 2012, **106**(6):1166-1170.
125. Imajoh M, Hashida Y, Nemoto Y, Oguri H, Maeda N, Furihata M, Fukaya T, Daibata M: **Detection of Merkel cell polyomavirus in cervical squamous cell carcinomas and adenocarcinomas from Japanese patients**. *Virol J* 2012, **9**:154.

126. Salehi-Vaziri M, Sadeghi F, Alamsi-Hashiani A, Haeri H, Monavari SH, Keyvani H: **Merkel cell polyomavirus and human papillomavirus infections in cervical disease in Iranian women**. *Archives of virology* 2015, **160**(5):1181-1187.
127. Antonsson A, Bialasiewicz S, Rockett RJ, Jacob K, Bennett IC, Sloots TP: **Exploring the prevalence of ten polyomaviruses and two herpes viruses in breast cancer**. *PLoS One* 2012, **7**(8):e39842.
128. Fimereli D, Gacquer D, Fumagalli D, Salgado R, Rothe F, Larsimont D, Sotiriou C, Detours V: **No significant viral transcription detected in whole breast cancer transcriptomes**. *BMC cancer* 2015, **15**:147.
129. Reza MA, Reza MH, Mahdiyeh L, Mehdi F, Hamid ZN: **Evaluation Frequency of Merkel Cell Polyoma, Epstein-Barr and Mouse Mammary Tumor Viruses in Patients with Breast Cancer in Kerman, Southeast of Iran**. *Asian Pacific journal of cancer prevention : APJCP* 2015, **16**(16):7351-7357.
130. Corbex M, Bouzbid S, Traverse-Glehen A, Aouras H, McKay-Chopin S, Carreira C, Lankar A, Tommasino M, Gheit T: **Prevalence of papillomaviruses, polyomaviruses, and herpesviruses in triple-negative and inflammatory breast tumors from algeria compared with other types of breast cancer tumors**. *PLoS One* 2014, **9**(12):e114559.
131. Goh S, Lindau C, Tiveljung-Lindell A, Allander T: **Merkel cell polyomavirus in respiratory tract secretions**. *Emerging infectious diseases* 2009, **15**(3):489-491.
132. Bialasiewicz S, Lambert SB, Whiley DM, Nissen MD, Sloots TP: **Merkel cell polyomavirus DNA in respiratory specimens from children and adults**. *Emerging infectious diseases* 2009, **15**(3):492-494.
133. Abedi Kiasari B, Vallely PJ, Klapper PE: **Merkel cell polyomavirus DNA in immunocompetent and immunocompromised patients with respiratory disease**. *J Med Virol* 2011, **83**(12):2220-2224.
134. Shikova E, Emin D, Alexandrova D, Shindov M, Kumanova capital A C, Lekov A, Moens U: **Detection of Merkel Cell Polyomavirus in Respiratory Tract Specimens**. *Intervirology* 2017, **60**(1-2):28-32.
135. Prezioso C, Di Lella FM, Rodio DM, Bitossi C, Trancassini M, Mele A, de Vito C, Antonelli G, Pietropaolo V: **Merkel Cell Polyomavirus DNA Detection in Respiratory Samples: Study of a Cohort of Patients Affected by Cystic Fibrosis**. *Viruses* 2019, **11**(6).
136. Bergallo M, Costa C, Terlizzi ME, Astegiano S, Curtoni A, Solidoro P, Delsedime L, Cavallo R: **Quantitative detection of the new polyomaviruses KI, WU and Merkel cell virus in transbronchial biopsies from lung transplant recipients**. *Journal of clinical pathology* 2010, **63**(8):722-725.
137. Babakir-Mina M, Ciccozzi M, Lo Presti A, Greco F, Perno CF, Ciotti M: **Identification of Merkel cell polyomavirus in the lower respiratory tract of Italian patients**. *J Med Virol* 2010, **82**(3):505-509.
138. Iaria M, Caccuri F, Apostoli P, Giagulli C, Pelucchi F, Padoan RF, Caruso A, Fiorentini S: **Detection of KI WU and Merkel cell polyomavirus in respiratory tract of cystic fibrosis patients**. *Clinical microbiology and infection : the official publication of the European Society of Clinical Microbiology and Infectious Diseases* 2015, **21**(6):603.e609-615.
139. Andres C, Ihrler S, Puchta U, Flaig MJ: **Merkel cell polyomavirus is prevalent in a subset of small cell lung cancer: a study of 31 patients**. *Thorax* 2009, **64**(11):1007-1008.
140. Joh J, Jenson AB, Moore GD, Rezazedeh A, Slone SP, Ghim SJ, Kloecker GH: **Human papillomavirus (HPV) and Merkel cell polyomavirus (MCPyV) in non small cell lung cancer**. *Experimental and molecular pathology* 2010, **89**(3):222-226.

141. Lasithiotaki I, Antoniou KM, Derdas SP, Sarchianaki E, Symvoulakis EK, Psaraki A, Spandidos DA, Stathopoulos EN, Siafakas NM, Sourvinos G: **The presence of Merkel cell polyomavirus is associated with deregulated expression of BRAF and Bcl-2 genes in non-small cell lung cancer**. *Int J Cancer* 2013, **133**(3):604-611.
142. Xu S, Jiang J, Yu X, Sheng D, Zhu T, Jin M: **Association of Merkel cell polyomavirus infection with EGFR mutation status in Chinese non-small cell lung cancer patients**. *Lung cancer (Amsterdam, Netherlands)* 2014, **83**(3):341-346.
143. Kim GJ, Lee JH, Lee DH: **Clinical and prognostic significance of Merkel cell polyomavirus in nonsmall cell lung cancer**. *Medicine* 2017, **96**(3):e5413.
144. Behdarvand A, Zamani MS, Sadeghi F, Yahyapour Y, Vaziri F, Jamnani FR, Nowruzi B, Fateh A, Siadat SD: **Evaluation of Merkel cell polyomavirus in non-small cell lung cancer and adjacent normal cells**. *Microbial pathogenesis* 2017, **108**:21-26.
145. Gheit T, Munoz JP, Levican J, Gonzalez C, Ampuero S, Parra B, Gaggero A, Corvalan AH, Meneses M, Tommasino M *et al*: **Merkel cell polyomavirus in non-small cell lung carcinomas from Chile**. *Experimental and molecular pathology* 2012, **93**(1):162-166.
146. Helmbold P, Lahtz C, Herpel E, Schnabel PA, Dammann RH: **Frequent hypermethylation of RASSF1A tumour suppressor gene promoter and presence of Merkel cell polyomavirus in small cell lung cancer**. *European journal of cancer (Oxford, England : 1990)* 2009, **45**(12):2207-2211.
147. Hourdequin KC, Lefferts JA, Brennick JB, Ernstoff MS, Tsongalis GJ, Pipas JM: **Merkel cell polyomavirus and extrapulmonary small cell carcinoma**. *Oncology letters* 2013, **6**(4):1049-1052.
148. Bhatia K, Modali R, Goedert JJ: **Merkel cell polyomavirus is not detected in mesotheliomas**. *Journal of clinical virology : the official publication of the Pan American Society for Clinical Virology* 2010, **47**(2):196-198.

Supplementary Table S2. MCPyV isolated in sewage water and environmental surfaces.

**Source**	**Sequence MCPyV genome**	**reference**
Sewage and river water (Brazil, Italy, Argentina, Uruguay)	Not done<br><br>M-cg-9-11.1<br>M-cg-9-11.3<br>M-cg-28-8.1<br>M-cg-28-8.16	[1-5]<br><br>[6]<br>[6]<br>[6]<br>[6]
Swimming pool	Not done	[7]
Environmental surfaces at lab and home (e.g. door handles, tv remote control, computer keyboard and mouse, etc)	Not done	[8]

References

[1] S. Bofill-Mas, J. Rodriguez-Manzano, B. Calgua, A. Carratala, R. Girones, Newly described human polyomaviruses Merkel cell, KI and WU are present in urban sewage and may represent potential environmental contaminants, Virol J, 7 (2010) 141.

[2] P. Di Bonito, S.D. Libera, S. Petricca, M. Iaconelli, L. Accardi, M. Muscillo, G. La Rosa, Frequent and abundant Merkel cell polyomavirus detection in urban wastewaters in Italy, Food and environmental virology, 7 (2015) 1-6.

[3] M. Rusinol, X. Fernandez-Cassi, N. Timoneda, A. Carratala, J.F. Abril, C. Silvera, M.J. Figueras, E. Gelati, X. Rodo, D. Kay, P. Wyn-Jones, S. Bofill-Mas, R. Girones, Evidence of viral dissemination and seasonality in a Mediterranean river catchment: Implications for water pollution management, Journal of environmental management, 159 (2015) 58-67.

[4] M. Iaconelli, S. Petricca, S.D. Libera, P. Di Bonito, G. La Rosa, First Detection of Human Papillomaviruses and Human Polyomaviruses in River Waters in Italy, Food and environmental virology, 7 (2015) 309-315.

[5] C. Torres, M.E. Barrios, R.V. Cammarata, D.M. Cisterna, T. Estrada, S. Martini Novas, P. Cahn, M.D. Blanco Fernandez, V.A. Mbayed, High diversity of human polyomaviruses in environmental and clinical samples in Argentina: Detection of JC, BK, Merkel-cell, Malawi, and human 6 and 7 polyomaviruses, The Science of the total environment, 542 (2016) 192-202.

[6] C. Torres, M.E. Barrios, R.V. Cammarata, M. Victoria, X. Fernandez-Cassi, S. Bofill-Mas, R. Colina, M.D. Blanco Fernandez, V.A. Mbayed, Phylodynamics of Merkel-cell polyomavirus and human polyomavirus 6: A long-term history with humans, Molecular phylogenetics and evolution, 126 (2018) 210-220.

[7] G. La Rosa, S. Della Libera, S. Petricca, M. Iaconelli, R. Briancesco, R. Paradiso, M. Semproni, P. Di Bonito, L. Bonadonna, First detection of papillomaviruses and polyomaviruses in swimming pool waters: unrecognized recreational water-related pathogens?, Journal of applied microbiology, 119 (2015) 1683-1691.

[8] V. Foulongne, V. Courgnaud, W. Champeau, M. Segondy, Detection of Merkel cell polyomavirus on environmental surfaces, J Med Virol, 83 (2011) 1435-143.

Supplementary Table S3. MCPyV NCCR variants and mutations compared to the consensus sequence.

**Variant (accession number)**	**Group**	**NCCR[a]**	**Source**	**Reference**
R06b (HM011538)	2	ins369AACTC	healthy facial skin	[1]
R09b (HM011539)	1	ΔT168	healthy facial skin	[1]
R10b (HM011540)	5	C51T, A251C, ins372ACAAC, Δ381-387AACAAGG, T449C	healthy facial skin	[1]
R12a (HM011541)	1	consensus	healthy facial skin	[1]
R12b (HM011542)	1	insA213	healthy facial skin	[1]
R13a (HM011543)	1	ΔAA221-222	healthy facial skin	[1]
R14a (HM011544)	2	ins369AACTC	healthy facial skin	[1]
R14b (HM011544)	2	ins369AACTC	healthy facial skin	[1]
R15a (HM011546)	4	Δ105-112CCTTAGAT	healthy facial skin	[1]
R15b (HM011547)	4	Δ105-112CCTTAGAT	healthy facial skin	[1]
R16b (HM011548)	6	A234T, ins373CAAC, insT379, insAA383, T449C	healthy facial skin	[1]
R17a (HM011555)	1	ΔT168	healthy facial skin	[1]
R17b (HM011556)	1	consensus	healthy facial skin	[1]
R18b (HM011550)	1	consensus	healthy facial skin	[1]
R20b (HM011551)	1	consensus	healthy facial skin	[1]
R25b (HM011552)	2	CT13T, ins369AAC	healthy facial skin	[1]
R26b (HM011553)	1	C272T, Δ392C	healthy facial skin	[1]
R30a (HM011557)	1	consensus	healthy facial skin	[1]
R30b (HM011554)	1	consensus	healthy facial skin	[1]
124/Sweden (KX827417)	1	consensus	blood AML patient	[2]
7673/2011/HUN (KC202810)	3	T102A, ins372TCAAT	MCC	[3]
915F 06 001 PJ2 (JQ479315)	1	consensus	healthy facial skin	[4]
915F 06 002 KN4 (JQ479316)	2	ins369AACTC	healthy facial skin	[4]
915F 06 005 FS2 (JQ479318)	1	consensus	healthy facial skin	[4]
915F 06 004 BG1 (JQ479317)	1	C6G	healthy facial skin	[4]
915F 06 007 FD3 (JQ479319)	1	consensus	healthy facial skin	[4]

915F 06 008 CG4 (JQ479320)	2	ins369AACTC	healthy facial skin	[4]
AiDo (KJ128376)	1	consensus	MCC	[5]
AmePI (KF266965)	1	ΔT379, insT401, T449C	healthy facial skin	[6]
BroLi (KJ128377)	1	consensus	MCC	[5]
CVG-1 (MH136801)	1	consensus	MCC	[7]
DyskA (KX781279)	1	consensus	swab from normal skin	[8]
EuroCauC1 (KF266963)	1	G363A	healthy facial skin	[6]
FraMerk2 (JN383838)	1	ΔG300	MCC	[9]
FraMerk20 (JN383839)	1	insA213, G301A, ΔA304, G309A, G363A	MCC	[9]
FraMerk22 (JN383840)	1	G143T, C170T, C176T, G188A, G363A	MCC	[9]
FraMerk24 (JN383841)	1	G96A, G300T	MCC	[9]
HB039C (KC571692)	6	A234T, ins373CAAC, insT379, insAA383, T449C	Feces children	unpublished[b]
HF (JF813003)	1	consensus	MCC	[10]
KIB (MK561422)	7	A234T, ins372TCAAC, ins38AA, ins429TTTCTCTTACAAAGGGAGGAGGACA	Blood healthy volunteer	[11]
LoKe (KJ128381)	1	consensus	MCC	[5]
M-cg-9-11.1 (MG241579)	2	ins369AACTC, insT401, T449C	sewage sample	[12]
M-cg-9-11.3 (MG241580)	2	Ins369AACTC	sewage sample	[12]
M-cg-28-8-1 (MG241581)	1	A361G	sewage sample	[12]
M-cg-28-8.16 (MG241582)	1	ΔA222	sewage sample	[12]
MCC85 (JF813002)	1	C372G	MCC	[10]
MCC339 (EU375804)	2	ins369AACTC	MCC	[13]
MCC344 (JF812999)	2	ins369AAC	MCC	[10]
MCC349 (JF813000)	1	consensus	MCC	[10]
MCC350 (EU375804)	1	G32C, G152T, G363A	MCC	[13]
MCC352 (JF813001)	1	consensus	MCC	[10]
MCVw156 (HM355825)	1	G32C, ΔA222	MCC	unpublished[c]
MKL-1 (FJ173815)	1	T52C	MCC	[14]
MKT-21 (FM865403)	2	ins369AACTC	MCC	[15]
MKT-22 (FM865404)	1	T94C, ΔT408, C448A	MCC	[15]
MKT-23 (FM864208)	2	G143A, C145T, C158T, A173G, ins369AACTC	MCC	[15]
MKT-26 (FM865405)	1	T86C, T210C, ΔA222, A435G	MCC	[15]
MKT-32 (FM995609)	1	T146C, ΔG274	MCC	[15]
MS-1 (JX045709)	2	ins369AACTC	MCC	[13]
OcepolW1 (KF266964)	5	T5G, C26A, G40A, T108G, ins372372ACAA, Δ381-387AACAAGG, C393T, T449C	Swab healthy skin	[6]
PeTa (KJ128378)	2	ins369AACTC	MCC	[5]

TKS (FJ464337)	6	A234T, ins373CAAC, insT379, insAA383, T449C	Kaposi's sarcoma	[16]
WaGa (KJ128379)	1	consensus	MCC	[5]
WoWe (KJ128380)	1	consensus	MCC	[5]

[a]The numbers refer to the nucleotide of the consensus NCCR
[b]direct submission by Li et al., 2013.
[c]direct submission by Carter JJ et al., 2010 and Cahill and Paulson, 2010.

References

1. Schowalter RM, Pastrana DV, Pumphrey KA, Moyer AL, Buck CB: **Merkel cell polyomavirus and two previously unknown polyomaviruses are chronically shed from human skin**. *Cell Host Microbe* 2010, **7**(6):509-515.
2. Song Y, Gyarmati P: **Identification of Merkel Cell Polyomavirus from a Patient with Acute Myeloid Leukemia**. *Genome announcements* 2017, **5**(3).
3. Horvath KB, Pankovics P, Battyani Z, Kalman E, Reuter G: **[A probable etiological role of Merkel cell polyomavirus in the development of Merkel cell carcinoma]**. *Orvosi hetilap* 2013, **154**(3):102-112.
4. Foulongne V, Sauvage V, Hebert C, Dereure O, Cheval J, Gouilh MA, Pariente K, Segondy M, Burguiere A, Manuguerra JC *et al*: **Human skin microbiota: high diversity of DNA viruses identified on the human skin by high throughput sequencing**. *PLoS One* 2012, **7**(6):e38499.
5. Schrama D, Sarosi EM, Adam C, Ritter C, Kaemmerer U, Klopocki E, Konig EM, Utikal J, Becker JC, Houben R: **Characterization of six Merkel cell polyomavirus-positive Merkel cell carcinoma cell lines: Integration pattern suggest that large T antigen truncating events occur before or during integration**. *Int J Cancer* 2019, **145**(4):1020-1032.
6. Martel-Jantin C, Filippone C, Tortevoye P, Afonso PV, Betsem E, Descorps-Declere S, Nicol JT, Touze A, Coursaget P, Crouzat M *et al*: **Molecular epidemiology of merkel cell polyomavirus: evidence for geographically related variant genotypes**. *J Clin Microbiol* 2014, **52**(5):1687-1690.
7. Velasquez C, Amako Y, Harold A, Toptan T, Chang Y, Shuda M: **Characterization of a Merkel Cell Polyomavirus-Positive Merkel Cell Carcinoma Cell Line CVG-1**. *Frontiers in microbiology* 2018, **9**:713.
8. Nguyen KD, Lee EE, Yue Y, Stork J, Pock L, North JP, Vandergriff T, Cockerell C, Hosler GA, Pastrana DV *et al*: **Human polyomavirus 6 and 7 are associated with pruritic and dyskeratotic dermatoses**. *J Am Acad Dermatol* 2016.
9. Martel-Jantin C, Filippone C, Cassar O, Peter M, Tomasic G, Vielh P, Briere J, Petrella T, Aubriot-Lorton MH, Mortier L *et al*: **Genetic variability and integration of Merkel cell polyomavirus in Merkel cell carcinoma**. *Virology* 2012, **426**(2):134-142.
10. Feng H, Kwun HJ, Liu X, Gjoerup O, Stolz DB, Chang Y, Moore PS: **Cellular and viral factors regulating Merkel cell polyomavirus replication**. *PLoS One* 2011, **6**(7):e22468.
11. Jin HT, Park SJ, Choi EK, Kim YS: **The frequency of Merkel cell polyomavirus in whole blood from immunocompetent and immunosuppressed patients with kidney disease and healthy donors**. *Microbial pathogenesis* 2019, **131**:75-80.
12. Torres C, Barrios ME, Cammarata RV, Victoria M, Fernandez-Cassi X, Bofill-Mas S, Colina R, Blanco Fernandez MD, Mbayed VA: **Phylodynamics of Merkel-cell polyomavirus and human polyomavirus 6: A long-term history with humans**. *Molecular phylogenetics and evolution* 2018, **126**:210-220.
13. Feng H, Shuda M, Chang Y, Moore PS: **Clonal integration of a polyomavirus in human Merkel cell carcinoma**. *Science (New York, NY)* 2008, **319**(5866):1096-1100.
14. Shuda M, Feng H, Kwun HJ, Rosen ST, Gjoerup O, Moore PS, Chang Y: **T antigen mutations are a human tumor-specific signature for Merkel cell polyomavirus**. *Proc Natl Acad Sci U S A* 2008, **105**(42):16272-16277.

15. Touze A, Gaitan J, Maruani A, Le Bidre E, Doussinaud A, Clavel C, Durlach A, Aubin F, Guyetant S, Lorette G *et al*: **Merkel cell polyomavirus strains in patients with merkel cell carcinoma**. *Emerging infectious diseases* 2009, **15**(6):960-962.
16. Katano H, Ito H, Suzuki Y, Nakamura T, Sato Y, Tsuji T, Matsuo K, Nakagawa H, Sata T: **Detection of Merkel cell polyomavirus in Merkel cell carcinoma and Kaposi's sarcoma**. *J Med Virol* 2009, **81**(11):1951-1958.

Supplementary Table S4. Putative consensus transcription factor binding motifs in the consensus MCPyV NCCR.

Transcription factor	Consensus motif	Motif in MCPyV NCCR	NCCR variants tested in this study with mutation in putative motif
AML1A	ACCACAAAAT	AAGTTGTGGT (238-247)*	
AP-1/c-JUN	TGACTCA	TGACTCA (287-293) and TGACTGA (314-320)	
AP-2αA	GCAGGC	GGAGGC (387-392)	10b (Δ381-387)
Cart-1	AATTA	AATTA (128-132)	
C/EBPα/β	TTGCTC	TTGCTG (266-271)	
Chx10	$^{A}/_{T}$T$^{A}/_{T}$GCTAATTA	TTTGCTAATTA (122-132)	
CUTL1	ATTGGT	ATTGCT (265-270)	
DP1	ATTTCCCGCC	GTTTCCCGCC (88-92)	
E2F-1	TCCCGCC	TCCCGCC (86-92)	
E2F-5	TTTCGCGC	TTTCCCGC (84-91)	
E47	ATCTGGC	TTCTGGC (278-284)	
EIIaE-A	CGCCCTC	CGCCCTT (89-95)	
Elk-1	GGAAG	GGAAG (359-363)	
ENKTF-1	TGGCG$^{C}/_{T}$$^{A}/_{T}$G	TGGCATTG (281-288)	
c-Ets-1	CTTCCTG	TTTCCTG (294-300)	
c-Ets-2	AGGAAATG	CATTTCCT (299-292)#	
FOXO3A	CTTAAATA	CTTAGATA (106-113)	
GATA-1	GATAAG	GATAAA (319-324)	
GATA-2	GCC$^{C}/_{T}$TATCT	AGATACTGC (109-117)	
HNF-3α	AAGTAAATAA	TTATTTATTT (458-449)	10b (T449C), 16b (T449C)
HOXD9	AATAAAAAATA	AACTTTTATT (267-258)	
LF-A1	TGCCCTG	TGCCCTT (102-108)	15a (Δ105-112); HUN (T102A)
NF1	TTGGC	GCCAA (208-204) and GCCAA (239-235)	
NF-AT	AATTTTTCC	TTTTTTTCC (162-179)	

NF-AT3	T^{A}/$_{T}$A^{A}/$_{T}$TTTTCC	TTTTTTTTCC (161-170)	
NF-AT4	A^{A}/$_{T}$TTATTTTTCC	ATTTATTTTTCA (451-462)	
NF-κB	TGGGAATTTCC	CTGCAGTTTCC (88-78)	
POU2F2C	ATGCAAAT	ATGCAGAAG (10-17)	
POU3F2	AAATGAA	AAATGAG (44-50)	
R2	TCCAAC	TCCAGA (168-173)	
RELA	CAGATTCCCC	CAGTTTCCCG (81-90)	
Sp1	G^{G}/$_{T}$GGGCGGG	CCCGCCCC (94-87)	
SRY	AAACAA^{A}/$_{T}$	AAACAAA (322-328)	
STAT1β	ATTTCCAAGA	ATTTCCTGGA (293-302)	
STAT4	ATTTCC	GTTTCC (83-88), TTTTCC (165-170), ATTTCC (293-298)	
STAT5A	GGAA	TTCC (85-88, 167-170, 295-298) and GGAA (359-362)	
TBP/TFIID	TATAAAAA	TTTTTATA (77-70)	
TFIIB	GTCTCAGA	GCCTCAGA (143-150)	
USF2	CAGGTGACC	AGTCACCTA (230-222)	
VDR	GGGT	GGGT (274-277)	
WT1	GTGTGTG	ACCTCAC (59-53)	

*Numbers refer to position in the consensus NCCR.

#On the complementary strand

The ALLGEN PROMO transcription factor prediction database was used [1,2]. Motifs with none, one (motif <6 base-pairs) or 2 (motifs >6 base-pairs) mutations compared to the consensus binding site in the MCPyV NCCR are shown.

References

[1] Messeguer X, Escudero R, Farré D, Núñez O, Martínez J, Albà MM. PROMO: detection of known transcription regulatory elements using species-tailored searches. Bioinformatics. 2002;18(2):333-4. DOI:10.1093/bioinformatics/18.2.333

[2] Farré D, Roset R, Huerta M, Adsuara JE, Roselló L, Albà MM, Messeguer X. Identification of patterns in biological sequences at the ALGGEN server: PROMO and MALGEN. Nucleic Acids Res. 2003;31(13):3651-3. DOI:10.1093/nar/gkg605
